# Grocery store interventions to change food purchasing behaviors: a systematic review of randomized controlled trials

**DOI:** 10.1093/ajcn/nqy045

**Published:** 2018-06-04

**Authors:** Jamie Hartmann-Boyce, Filippo Bianchi, Carmen Piernas, Sarah Payne Riches, Kerstin Frie, Rebecca Nourse, Susan A Jebb

**Affiliations:** Nuffield Department of Primary Care Health Sciences, University of Oxford, Oxford, United Kingdom

**Keywords:** grocery stores, food purchasing, diet, behavior change, systematic review

## Abstract

**Background:**

Diet is an important determinant of health, and food purchasing is a key antecedent to consumption.

**Objective:**

We set out to evaluate the effectiveness of grocery store interventions to change food purchasing, and to examine whether effectiveness varied based on intervention components, setting, or socioeconomic status.

**Design:**

We conducted a systematic review of randomized controlled trials (search performed June 2017). Studies must have: aimed to change food purchasing; been implemented in grocery stores (real or simulated); reported purchasing; and had a minimal control or compared interventions fulfilling our criteria. Searching, screening, bias assessment, and data extraction followed Cochrane methods. We grouped studies by intervention type (economic, environmental, swaps, and/or education), synthesized results narratively, and conducted an exploratory qualitative comparative analysis.

**Results:**

We included 35 studies representing 89 interventions, >20,000 participants, and >800 stores. Risk of bias was mixed. Economic interventions showed the most promise, with 8 of the 9 studies in real stores and all 6 in simulated environments detecting an effect on purchasing. Swap interventions appeared promising in the 2 studies based in real stores. Store environment interventions showed mixed effects. Education-only interventions appeared effective in simulated environments but not in real stores. Available data suggested that effects of economic interventions did not differ by socioeconomic status, whereas for other interventions impact was variable. In our qualitative comparative analysis, economic interventions (regardless of setting) and environmental and swap interventions in real stores were associated with statistically significant changes in purchasing in the desired direction for ≥1 of the foods targeted by the intervention, whereas education-only interventions in real stores were not.

**Conclusions:**

Findings suggest that interventions implemented in grocery stores—particularly ones that manipulate price, suggest swaps, and perhaps manipulate item availability—have an impact on purchasing and could play a role in public health strategies to improve health. Review protocol registered at https://www.crd.york.ac.uk/PROSPERO/ as CRD42017068809.

## INTRODUCTION

Food purchasing is a key antecedent of food consumption, and interventions in grocery stores are of interest to those trying to change food purchasing to promote health and those concerned with the marketing and sales of foods and drinks (1). The goals of each may differ but the types of interventions are similar. These include economic interventions, such as financial incentives and/or disincentives (2), environmental interventions, which could work at the conscious or unconscious level (3), and education, or combinations of the above. Evaluating the effectiveness of these interventions is complex. Testing such interventions in real grocery stores is not always feasible, and thus some of the aforementioned strategies have been evaluated within simulated (e.g., virtual) stores, with potential for different effects. In addition, interventions to change food purchasing may attenuate or exacerbate health disparities. Socioeconomically disadvantaged populations are more likely to suffer from nutrition-related morbidity, and there is some evidence to suggest that certain interventions, particularly those relying on executive functioning, may be more effective in more socioeconomically advantaged groups (4–7). On the other hand, disadvantaged groups may be more sensitive to economic interventions (8).

Previous systematic reviews of grocery store interventions are either now outdated (9) or more narrow in scope than ours [e.g., focus exclusively on interventions designed to promote health (9–11), or do not include price or labeling (10), or were conducted in specific populations (11)], which restricts the ability of researchers and policy-makers to develop a comprehensive picture of the extant evidence. Here we focus on randomized controlled trials (RCTs) that evaluated the effectiveness of interventions implemented in grocery stores to change purchasing behavior and consumption, with no restrictions by intervention or population type. We also set out to examine how, if at all, effectiveness varied based on intervention components, setting (real compared with simulated) and socioeconomic status (SES).

Our aim was to understand the effectiveness of interventions in grocery stores to aid the development of strategies to improve public health and to reduce inequalities, and to identify evidence gaps.

## METHODS

A protocol was published in advance and is available in PROSPERO (https://www.crd.york.ac.uk/PROSPERO/; CRD42017068809) (12). Methods for searching, screening, data extraction, and quality assessment followed those set out in the Cochrane handbook (13).

### Searching and inclusion criteria

We searched 13 electronic databases on 2 June 2017 using terms relating to grocery stores, food and nonalcoholic beverages, purchase and choice behaviors, and randomized controlled trials [see protocol (12) for full strategy; **Supplemental Table 1** for MEDLINE search strategy]. We also screened reference lists of included studies.

We included RCTs when interventions were designed to change the purchase of any foods, nonalcoholic drinks, nutrients, energy, or products belonging to a defined dietary pattern or with defined dietary scores and when purchases of any of the above were reported at the individual or store level (our primary outcome). Our secondary outcome was participants’ consumption of the above items. To be included, interventions must have been implemented partly or completely in any online or physical grocery store, including simulated stores. Studies must have had a minimal control or a comparison between ≥2 interventions fulfilling the aforementioned criteria.

### Screening, data extraction, and risk of bias assessment

Two reviewers independently screened studies for inclusion at title/abstract and full-text stage, extracted data with the use of a predefined and prepiloted data-extraction form, and assessed risk of bias with the use the Cochrane risk-of-bias tool (13), with discrepancies resolved by discussion or referral to a third reviewer. Data were extracted on: recruitment methods; inclusion/exclusion criteria; population; setting; intervention and comparator characteristics; outcomes; and whether these varied by socioeconomic status. When needed, we contacted authors for further information via email.

### Analysis

We conducted a narrative synthesis of the data, tabulating results for our primary and secondary outcomes from the original study reports. When multiple time points were available, we chose that measured during or as close as possible to the end of the intervention. We did not conduct a meta-analysis due to substantial clinical heterogeneity with regards to the reported outcome, outcome measures, and study designs. We classified interventions into one of 4 categories, informed by existing literature (2, 3, 14):

- Economic interventions [any intervention including a price increase, decrease, or financial reward (2)].
- Store environment changes [any intervention involving changes to the microenvironment (3), but not including economic interventions which are covered by (A), swaps which are covered by (C) or interventions based on product labeling or consumer education alone which are covered by (D)].
- Swap interventions, which offer consumers the opportunity to replace their usual food with a healthier alternative [but not including economic interventions, which are covered by (A)].
- Labeling and/or educational interventions [interventions involving product labeling (14) and consumer education/information, but not economic or other store environment changes].

We present results separately for real and simulated settings.

### Qualitative comparative analysis 

In addition to our narrative synthesis, we also employed an exploratory crisp-set qualitative comparative analysis (QCA) (15) to identify combinations of intervention components associated with statistically significant changes (*P* < 0.05) in the desired direction for at least one of the foods targeted by the intervention. We used 5 variables in our QCA. The first 4 were modelled on the groups above but were not mutually exclusive, namely whether or not the intervention involved an economic component (as per group A) or changes to the store environment (as per B) or swaps (as per C) or consumer education/information (as per D). The fourth variable was whether or not the intervention was based in a real grocery store. We only included comparisons between eligible interventions and minimal controls. We excluded configurations that originated from multiple similar interventions tested in one single study (16, 17). QCA is a method that aims to identify variables present when an intervention is effective. The analyses were conducted with the use of fsQCA software, with a consistency threshold of 0.75 above and a frequency threshold of 2. Accordingly, a combination of intervention characteristics was defined as being associated with “significant changes in the desired direction for at least one of the foods targeted by the intervention” when ≥75% of all interventions with this combination, and ≥2 such interventions, were associated with the aforementioned outcome. Though prespecified in our protocol, this was an exploratory analysis used to augment the narrative review.

## RESULTS

### Search and screening

Excluding duplicates, 1466 references were retrieved from database searches, with 1 additional paper from screening reference lists. We assessed the full text of 135 studies, 100 of which were excluded, most commonly because the study was not an RCT or did not measure purchasing behavior (**Supplemental Figure 1**). We included 35 studies, representing 55 references.

### Characteristics of included studies

The key characteristics of included studies are summarized in **Table 1** and described below, with more detail in **Supplemental Tables 1 and 2**.

**TABLE 1:** Key characteristics of included studies^1^

Study ID	Country	Participants, *n*	Stores, *n*	Store type	SES	Intervention group^2^	Control group
Achabal et al. (18)	USA	NR	372	Real: supermarket	NR	D (arms 1 and 2)	Yes
Anderson et al. (19)	USA	104	2	Real: supermarket	Mean income lower than average	A (arm 1)	Yes
Ball et al. (20)	Australia	437	NR	Real: supermarket	∼40% low SES	A (arms 1 and 2)	Yes
Ball et al. (21)	USA	211	2	Real: supermarket	Recruited disadvantaged	D (arm 1)	Yes
Brimblecombe et al. (22)	Australia	NR	20	Real: supermarket	NR	A (arms 1 and 2)	No
Budd et al. (23)	USA	NR	24	Real: corner/convenience store	Recruited from low SES	A (arms 1 and 3); B (arm 2)	Yes
Dhar and Hoch (24)	USA	NR	86	Real: supermarket	NR	A (arms 1 and 2)	No
Dreze et al. (25)	USA	NR	60	Real: supermarket	NR	B (arms 1 and 2)	Yes
Ducrot et al. (16)	France	11,981	1	Simulated (Web-based)	Range, but mainly mid to high	D (arms 1–4)	Yes
Elofsson et al. (26)	Sweden	NR	17	Real: supermarket and corner/convenience	NR	B (arms 1 and 2)	No
Epstein et al. (27)	USA	199	1	Simulated (Web-based)	Range, but mainly mid to high	A (arms 1–4)	Yes
Forwood et al. (17)	UK	720	1	Simulated (Web-based)	Evenly distributed across IMD quintiles	C (arms 1–4)	Yes
Foster et al. (28)	USA	NR	8	Real: supermarket	Low to moderate income census tract	B (arm 1)	Yes
Geliebter et al. (29)	USA	47	2	Real: supermarket	NR	A (arm 1)	Yes
Huang et al. (30)	Australia	497	1	Real: online supermarket	Majority high	C (arm 1)	Yes
Jeffery et al. (31)	USA	NR	8	Real: supermarket	NR	B (arm 1)	Yes
Kristal et al. (32)	USA	960	8	Real: supermarket	NR	A (arm 1)	Yes
Lent et al. (33)	USA	767 (children)	24	Real: corner/convenience store	Low income area	B (arm 1)	Yes
Ma et al. (34)	China	NR	129	Real: supermarket and corner/convenience	NR	A (arm 1); B (arm 2)	Yes
Milliron et al. (35)	USA	153	1	Real: supermarket	Median percent federal poverty guideline 300%	B (arms 1 and 2)	No
Nederkoorn et al. (36)	Netherlands	306	1	Simulated (Web-based)	Range, but mainly mid to high	A (arm 1)	Yes
Ni Mhurchu et al. (37)	New Zealand	830	8	Real: supermarket	Range, but mainly mid to high	A (arm 1)	Yes
Ni Mhurchu et al. (38)	New Zealand	1357	NR	Real: supermarket	Range, but mainly mid to high	D (arms 1 and 2)	Yes
Phipps et al. (39)	USA	58	1	Real: supermarket	Majority low	A (arm 1)	Yes
Russo et al. (40)	USA	NR	14	Real: supermarket	NR	B (arms 1–12)	Yes
Smith et al. (41)	New Zealand	151	NR	Real: supermarket	Low income	A (arm 1)	Yes
Thorndike et al. (42)	USA	575	6	Real: corner/convenience store	Low income	B (arm 1)	Yes
Wansink et al. (43)	Canada	169	1	Real: supermarket	NR	B (arms 1–6)	No
Waterlander et al. (44)	Netherlands	115	1	Simulated (physical)	Range, but majority mid or high	A (arm 1)	Yes
Waterlander et al. (45)	Netherlands	117	1	Simulated (Web-based)	Low SES	A (arm 1)	No
Waterlander et al. (46)	Netherlands	39	1	Simulated (Web-based)	Range, but majority mid or high	A (arm 1)	No
Waterlander et al. (47)	Netherlands	151	4	Real: supermarket	Low SES	A (arm 1)	Yes
Waterlander et al. (48)	Netherlands	95	1	Simulated (Web-based)	Majority low SES	A (arm 1)	Yes
Winett et al. (49)	USA	40	NR	Real: supermarket	Range, but majority mid or high	D (arm 1)	Yes
Winett et al. (50)	USA	77	1	Real: supermarket	NR	C (arm 1)	Yes

^1^ID, identification; NR, not reported; SES, socioeconomic status.

^2^(A) Economic interventions (any intervention including a price increase, decrease, or financial reward); (B) store environment changes [any intervention involving changes to the microenvironment, but not including economic interventions, which are covered by (A), swaps, which are covered by (C), or interventions based on product labeling or consumer education alone, which are covered by (D)]; (C) swap interventions, which offer consumers the opportunity to replace their usual food with a healthier alternative [but not including economic interventions, which are covered by (A)]; (D) labeling and/or educational interventions (interventions involving product labeling and/or consumer education/information, but not economic or other store environment changes).

### Participants and settings

Twenty-two studies randomized at the individual level. The remainder randomized at the store or community level, 10 of which did not report the number of participants/customers included. Across those studies that reported it, the number of participants included in this review was 20,156. One study was conducted in children (mean age 11 y). When reported, mean age across studies in adults ranged from 29 to 52 y (median 42 y) (33). Nine studies reported BMI (in kg/m^2^); when reported, means ranged from 25.8 to 30.2. In the 23 studies that reported gender, all were predominantly female (range 55–100%, median 81%). In the 15 studies that reported ethnicity, 11 had a majority of white/Caucasian participants. Twelve studies had inclusion criteria or recruitment settings that specifically targeted people from socioeconomically disadvantaged groups. When reported, the remainder (11 studies) included people predominantly of middle or high SES.

Eighteen studies were conducted in the United States, 6 in the Netherlands, 3 in Australia and New Zealand, and 1 each in Canada, China, France, Sweden, and the United Kingdom. Across those studies that reported store number, 807 stores were included. Nineteen studies described study area: 10 were conducted in urban/metropolitan settings; 2 in suburban settings; 3 across mixed settings; and 4 in rural areas.

Table 1 and Supplemental Table 1 contain more detail.

### Interventions and comparators

Twenty-seven of the studies consisted of interventions conducted in functioning grocery stores that existed outside of the research context: 21 exclusively in physical supermarkets; 3 exclusively in convenience/corner stores; 2 in supermarkets and convenience/corner stores; and 1 in an online supermarket. The remaining 8 studies were conducted in simulated supermarkets. Overall, the 35 included studies represented 89 intervention arms and 28 control arms (no intervention) that met our inclusion criteria, with 57 intervention versus control comparisons. The vast majority of interventions (81 of 89) were implemented solely in the store environment.

Thirty-one of 35 studies aimed to promote health, whereas 2 aimed to increase store profit, 1 aimed to increase the volume of food purchased, and 1 aimed to increase sales of a specific (nonhealth-related) product. Intervention length ranged from a one-off shopping trip to 2 y. Interventions typically consisted of multiple components, which are summarized below (for more detail see Table 1 and Supplemental Table 2).

Of the 89 interventions, 43 were economic interventions (group A). Of these, 13 involved price increases, 35 involved price decreases, and 1 involved financial rewards that were received post-shopping (39). Eighteen also involved advertising or signage in-store, 17 also provided education or information to consumers, and 2 each also involved in-store taste testing and changes to item stocking levels.

A further 30 interventions involved changes to the store environment with no economic components (group B). Twenty-one involved signage, 5 altered item placement, and 13 involved other changes, including partitioned grocery carts, and providing convenience stores with additional refrigerated units for produce. Twenty-seven also provided education or information to consumers.

A further 6 interventions involved suggested swaps (group C), either as a standalone intervention (17) or along with additional educational components (30, 50).

The remaining 10 interventions consisted of consumer education or information, or product labeling, without any additional economic or store environment changes (group D). Six of these evaluated different forms of product labeling; the remainder provided educational information (typically in the form of print leaflets) to participants.

### Outcomes

Our primary outcome was purchasing behavior. Twenty-nine studies measured purchasing during the intervention and for the remaining 6 we used immediately postintervention data. Twenty-five studies reported purchases at the individual level and 11 at the store level. Outcome was measured objectively (typically via sales data or transaction data) in all studies except 5 in which a self-reported measure was used (Supplemental Table 2). Five also reported on consumption as an outcome, typically via dietary questionnaires.

### Risk of bias

Ten studies were judged to be at low risk of bias across all domains assessed (i.e., at low risk of bias overall) and 14 at high risk of bias in ≥1 of the domains assessed (i.e., at high risk of bias overall). The remaining studies are considered at unclear risk of bias. **Table 2** lists judgments by domain for individual studies.

**TABLE 2:** Risk of bias judgments^1^

Study ID	Random sequence generation	Allocation concealment	Blinding of outcome assessors	Attrition	Other^2^	Overall
Achabal et al. (18)	Unclear	Unclear	Low	Low	NA	Unclear
Anderson et al. (19)	Unclear	Unclear	Low	Unclear	NA	Unclear
Ball et al. (20)	Low	Low	Low	Low	NA	Low
Ball et al. (21)	Low	Low	Low	Low	NA	Low
Brimblecombe et al. (22)	Unclear	Low	Low	Low	High	High
Budd et al. (23)	Low	Low	High	Low	NA	High
Dhar and Hoch (24)	Unclear	Unclear	Low	Low	NA	Unclear
Dreze et al. (25)	Unclear	Unclear	Low	Low	NA	Unclear
Ducrot et al. (16)	Low	Low	Low	High	NA	High
Elofsson et al. (26)	Unclear	Low	Low	Low	NA	Unclear
Epstein et al. (27)	Unclear	Low	Low	Low	NA	Unclear
Forwood et al. (17)	Low	Low	Low	High	NA	High
Foster et al. (28)	Unclear	Unclear	Low	Low	NA	Unclear
Geliebter et al. (29)	Low	Low	Low	High	NA	High
Huang et al. (30)	Low	Low	Low	Low	NA	Low
Jeffery et al. (31)	Unclear	Unclear	Low	Low	NA	Unclear
Kristal et al. (32)	Unclear	Unclear	High	High	NA	High
Lent et al. (33)	Unclear	Unclear	High	Low	NA	High
Ma et al. (34)	Low	Low	Low	Low	NA	Low
Milliron et al. (35)	Low	Low	Unclear	Low	NA	Unclear
Nederkoorn et al. (36)	Unclear	Unclear	Low	Unclear	NA	Unclear
Ni Mhurchu et al. (37)	Low	Low	Low	Low	NA	Low
Ni Mhurchu et al. (38)	Low	Low	Low	Low	NA	Low
Phipps et al. (39)	Low	Low	Low	Low	NA	Low
Russo et al. (40)	Unclear	Unclear	Low	Low	NA	Unclear
Smith et al. (41)	Low	Low	Low	Low	NA	Low
Thorndike et al. (42)	Unclear	Unclear	Low	High	NA	High
Wansink et al. (43)	Unclear	High	High	Low	NA	High
Waterlander et al. (44)	Low	Unclear	Low	High	NA	High
Waterlander et al. (45)	Low	Low	Low	Low	NA	Low
Waterlander et al. (46)	Unclear	Low	Unclear	High	NA	High
Waterlander et al. (47)	Low	Unclear	Low	High	NA	High
Waterlander et al. (48)	Low	Low	Low	Low	NA	Low
Winett et al. (49)	Unclear	Unclear	High	High	High	High
Winett et al. (50)	Unclear	Unclear	Low	Unclear	High	High

^1^ID, identification; NA, not available.

^2^This category includes examples for high risks of bias such as incomplete implementation of the intervention (22), and inappropriate exclusion of participants in the final results (49, 50).

### Effects of interventions

The effects of the interventions on our primary and secondary outcomes are summarized by group. No studies reported statistically significant results for our primary or secondary outcomes that were in the direction opposite from that intended. **Tables 3** and **4** contain numeric data (when available) for the results presented below; **Supplemental Table 3** provides further data on relevant outcomes at our primary time point.

**TABLE 3:** Effects of interventions on purchasing behavior: numeric data for outcomes presented in text^1^

Study ID	Outcome	Comparison	Between-group difference	*P* value	Group^2^
Achabal et al. (18)	Aggregate purchases over 6 target items	3-way comparison (ANOVA)	NR	0.505	D
Anderson et al. (19)	Individual purchases of fat (g)	Intervention vs. control	−0.173	<0.05	A
	Individual purchases of fiber (g)	Intervention vs. control	0.184	<0.01	A
	Individual purchases of fruit and vegetables (g)	Intervention vs. control	0.198	<0.01	A
Ball et al. (20)	Total vegetable (g/wk)	Intervention vs. control	232.7 (3.8, 461.6)^3^	0.046	A
Ball et al. (21)	Total vegetable (g/wk)	Intervention vs. control	19.24 (−158.21, 196.70)	0.832	D
	Total fruit (g/wk)	Intervention vs. control	−10.06 (−176.65, 156.52)	0.906	D
Brimblecombe et al. (22)	Total vegetable (g)	Discount + environment vs. discount alone	Percentage change 13.6% (2.6%, 25.7%)	0.014	A
Budd et al. (23)	Sales (units) of promoted snack foods (phase 3)	Intervention 1 (pricing) vs. control	3.6 ± 18.8^4^	>0.05	A
	Sales (units) of promoted snack foods (phase 3)	Intervention 2 (communication) vs. control	2.9 ± 8.0	>0.05	B
	Sales (units) of promoted snack foods (phase 3)	Intervention 3 (both combined) vs. control	6.4 ± 13.9	<0.05	A
Dhar and Hoch (24)	Sales of ready-to-eat cereal (units)	Intervention 1 (coupons) vs. intervention 2 (bonus buys)	NR	>0.05	A
Dreze et al. (25)	Sales of bottled juices ($ sales)	Intervention 1 (space to movement) vs. control	+4.9%	<0.001	B
		Intervention 2 (product reorganization) vs. control	NR	NR^5^	
	Sales of canned soup ($ sales)	Intervention 1 (space to movement) vs. control	+6.3%	<0.001	
		Intervention 2 (product reorganization) vs. control	−6%	<0.05	
	Sales of canned seafood ($ sales)	Intervention 1 (space to movement) vs. control	−1%	0.09	
		Intervention 2 (product reorganization) vs. control	NR	NR	
	Sales of frozen entrees ($ sales)	Intervention 1 (space to movement) vs. control	+4.4%	<0.001	
		Intervention 2 (product reorganization) vs. control	NR	NR	
	Sales of refrigerated juices ($ sales)	Intervention 1 (space to movement) vs. control	+2.6%	<0.001	
		Intervention 2 (product reorganisation) vs. control	NR	NR	
Ducrot et al. (16)	Overall nutritional quality (FSA/100 g)	Intervention 1 (5-colour nutrition label) vs. control	NR	<0.05	D
		Intervention 2 (multiple traffic lights) vs. control	NR	<0.05	
		Intervention 3 (green ticks) vs. control	NR	<0.05	
		Intervention 4 (guideline daily amounts) vs. control	NR	NS	
Elofsson et al. (26)	Sales of climate-certified milk (log)^6^	Intervention vs. control	Percentage difference 6.33% ± 0.029%	<0.05	B
Epstein et al. (27)	Total energy (kcal) from subsidized food	Subsidies vs. control	13.74 (8.51, 18.97)	<0.001	A
	Total energy (kcal) from subsidized food	Taxes vs. control	−6.61 (−11.94, −1.28)	0.02	
	Total energy (kcal)	Subsidies vs. control	−14.37 (−33.54, 4.81)	0.14	
	Total energy (kcal)	Taxes vs. control	−17.68 (−37.03, 1.68)	0.07	
Forwood et al. (17)	Total basket energy density (kJ/100 g)	All interventions vs. control	−24.1 (4.04, −52.23)	NS	C
Foster et al. (28)	Skimmed milk (oz)	Intervention vs. control	Mean difference in change pre/post 1509.1 ± 1079.9	0.0078	B
	Frozen chicken nuggets (units)	Intervention vs. control	Mean difference in change pre/post 20.5 ± 10.4	0.0074	
	In-aisle water (oz)	Intervention vs. control	Mean difference in change pre/post 1690.0 ± 6649.8	0.0109	
	Checkout water (units)	Intervention vs. control	Mean difference in change pre/post 18.5 ± 6.0	0.0002	
Geliebter et al. (29)	Weekly purchases of fruit and vegetables (unit not specified)	Intervention vs. control	NR	<0.001	A
Huang et al. (30)	Saturated fat (% from energy)	Intervention vs. control	−0.66 (0.48, 0.84)	<0.001	C
Jeffery et al. (31)	Weekly average sales of low-fat frozen desserts	Intervention vs. control	Increased	NS	B
	Weekly average sales of low-fat cottage cheese	Intervention vs. control	Increased	NS	
Kristal et al. (32)	Fruit and vegetable purchases at 1 y (% purchasing fruit or vegetable on day interviewed)	Intervention vs. control	NR. At baseline, 71.6% intervention and 70.4% control. At 1 y, 80.3% intervention and 78.7% control	>0.05	A
Lent et al. (33)	Energy (kcal)	Control vs. intervention	0.88 (0.5, −1.5)	0.58	B
	Fat (g)	Control vs. intervention	0.77 (0.5, −1.3)	0.32	
	Sodium (mg)	Control vs. intervention	1.21 (0.7, −2.2)	0.53	
	Carbohydrates (g)	Control vs. intervention	1.21 (0.7, −2.1)	0.50	
	Sugars (g)	Control vs. intervention	0.84 (0.4, −1.6)	0.61	
	Protein (g)	Control vs. intervention	1.17 (0.7, −2.1)	0.60	
	Fiber (g)	Control vs. intervention	0.78 (0.5, −1.5)	0.45	
Ma et al. (34)	Monthly sales of salt substitute (kg)	Intervention 1 (price subsidy and health education) vs. control	35.80 (21.54, 50.06)	<0.001	A
	Monthly sales of salt substitute (kg)	Intervention 2 (health education) vs. control	16.99 (2.66, 31.33)	0.020	B
Milliron et al. (35)	Fruit servings (g/1000 kcal)	Intervention vs. control	NR	0.002	B
	Dark green/yellow vegetables (servings/1000 kcal)	Intervention vs. control	NR	0.034	
Nederkoorn et al. (36)	Total energy (kcal)	Intervention vs. control	0.021	<0.01	A
Ni Mhurchu et al. (37)	Saturated fat (% from total energy)	Intervention vs. control	−0.02% (−0.40, 0.36)	0.91	A
	Predefined healthier foods (kg/wk)	Intervention vs. control	0.79 (0.43, 1.16)	<0.001	
Ni Mhurchu et al. (38)	All foods (nutrient profile score)	Intervention 1 (traffic light labels) vs. control	0.08 (−0.38, 0.54)	0.74	D
		Intervention 2 (health star rating) vs. control	−0.22 (−0.68, 0.25)	0.36	
Phipps et al. (39)	Fruit and vegetables (servings/wk)	Intervention vs. control	10.2 (3.6, 25.7)	<0.001	A
Russo et al. (40)	Overall nutritional quality (across all product categories)	Interventions vs. control	−0.029	NS	B
Smith et al. (41)	Total food expenditure ($NZ)	Intervention vs. control	15.20 (1.46, 28.94)	0.030	A
Thorndike et al. (42)	Store sales of WIC fruit and vegetables	Intervention vs. control	15.20	0.030	B
Wansink et al. (43)	Fruit and vegetable expenditure ($)^5^	Comparing: control cart, 35% partition cart, and 50% partition cart	*F* 10.15	<0.01	B
		Comparing: health/nutrition flyer compared to value/cost-savings flyer	*F* 72.66	<0.01	
		Comparing: in health/nutrition conditions, control cart, the 35% partition cart, and the 50% partition cart	NR	<0.05	
		Comparing: in value/cost-savings conditions, control cart, the 35% partition cart, and the 50% partition cart	NR	>0.05	
Waterlander et al. (44)	Total fruit and vegetables (number of items)	Intervention vs. control	1.33 (−0.16, 2.82)	0.08	A
Waterlander et al. (45)	Healthy foods (number)	Intervention 1 (50% discount) vs. control	6.62 (2.47, 10.78)	<0.01	A
	Total calories purchased	Intervention 1 (50% discount) vs. control	10,505 (4376, 16,635)	<0.01	
Waterlander et al. (46)	Healthy foods (number)	Intervention 1 (10% discount) vs. Intervention 2 (50% discount)	−8.58 (−13.4, −3.75)	<0.01	A
	Total calories purchased	Intervention 1 (10% discount) vs. Intervention 2 (50% discount)	Authors state “the discounts lead to an increased amount of energy purchased”	NR	
Waterlander et al. (47)	Fruit and vegetables (kg)	Intervention 1 (discount) vs. control	5252 (2836, 7668)	<0.001	A
Waterlander et al. (48)	Sugar-sweetened beverages (L)	Intervention vs. control	−0.90 (−1.70, −0.10)	<0.05	A
Winett et al. (49)	Simple carbohydrates (% energy)	Intervention vs. control	*F* 2.09 (% change intervention +5.1, control −0.0)	<0.05	D
Winett et al. (50)	High-fat meat (units)	Intervention vs. control	*F* 14.87 (equates to ∼37% decrease)	<0.001	C
	High-fiber grains/cereals (units)	Intervention vs. control	*F* 11.95 (equates to ∼62% increase)	<0.001	
	High-fat dairy (units)	Intervention vs. control	*F* 4.53 (equates to ∼20% decrease)	<0.05	

^1^Between-group differences are given as the β statistic unless indicated otherwise. Data here are those extracted from published studies; no additional calculation was conducted. Readers are encouraged to look to the full study reports for more information on outcome data (e.g., by individual arm or at different follow-up points). *P* values shown as reported in the published studies. CCF, Climate Certification of Food; FSA, Food Standard Agency scores; ID, identification; NR, not reported; WIC, Special Supplemental Program for Women, Infants, and Children; $NZ, New Zealand dollars.

^2^(A) Economic interventions (any intervention including a price increase, decrease, or financial reward); (B) store environment changes [any intervention involving changes to the microenvironment, but not including economic interventions, which are covered by (A), swaps, which are covered by (C), or interventions based on product labeling or consumer education alone, which are covered by (D)]; (C) swap interventions, which offer consumers the opportunity to replace their usual food with a healthier alternative [but not including economic interventions, which are covered by (A)]; (D) labeling and/or educational interventions (interventions involving product labeling and/or consumer education/information, but not economic or other store environment changes).

^3^Data in parentheses are 95% CIs (all such values).

^4^Mean ± SE (all such values).

^5^Factorial trial; individual intervention vs. control comparisons not presented in study report.

^6^Climate certified according to the Swedish standards for Climate Certification of Food (CCF). The CCF is a voluntary labeling scheme that requires certified food producers to strive towards a significant reduction of greenhouse gas emissions by focussing on the production choices with the largest climate impact (26).

**TABLE 4:** Effects of interventions on consumption: numeric data for outcomes presented in text^1^

Study ID	Outcome	Comparison	Between-group difference^1^	*P* value	Group^2^
Ball et al. (20)	Total vegetable (g/wk)	Intervention vs. control	−25.8	0.672	A
Ball et al. (21)	Total vegetable (servings/d)	Intervention vs. control	0.49	<0.001	D
	Total fruit (servings/d)	Intervention vs. control	−0.05	0.666	
Geliebter et al. (29)	Intake of fruit and vegetables (g/d)	Intervention vs. control	NR	NS	A
Kristal et al. (32)	Fruit and vegetable intake at 1 y (servings/d)	Intervention vs. control	NR. At baseline, mean 3.21 ± 1.75 intervention, 3.14 ± 1.74 control. At 1 y, mean 3.54 ± 1.79 intervention, 3.44 ± 1.83 control	>0.05	A
Waterlander et al. (47)	% participants who consumed sufficient (≥400 g/d) amount of fruit and vegetables	Intervention vs. control	NR. Authors state: “The percentage of participants who consumed sufficient amounts of F&Vs increased significantly from 42.5% at baseline to 61.3% at 6 mo in the discount groups (*P* = 0.03). For the nondiscount groups, these percentages were 52.7% and 52.5%, respectively (*P* = 0.80)”	NR	A

^1^Between-group differences reported as β statistic or mean ± SD unless indicated otherwise. *P* values shown as reported in the published studies. F&V, fruit and vegetable; ID, identification; NR, not reported.

^2^(A) Economic interventions (any intervention including a price increase, decrease, or financial reward); (B) store environment changes [any intervention involving changes to the microenvironment, but not including economic interventions, which are covered by (A), swaps, which are covered by (C), or interventions based on product labeling or consumer education alone, which are covered by (D)]; (C) swap interventions, which offer consumers the opportunity to replace their usual food with a healthier alternative [but not including economic interventions, which are covered by (A)]; (D) labeling and/or educational interventions (interventions involving product labeling and/or consumer education/information, but not economic or other store environment changes).

### Group A: economic interventions

Twelve studies tested economic interventions in real store environments; 11 applied discounts on target items at time of purchase and 1 provided store vouchers after purchase (39). Four also reported consumption as an outcome.

#### Physical stores

##### Intervention compared with control

All but 1 of the 9 studies comparing price decreases with control detected a statistically significant increase in purchases for ≥1 of the target items (see Table 3); none of the studies that decreased prices of healthy food reported increases in purchases of unhealthy items. Both Anderson et al. (19) and Ni Mhurchu et al. (37), reporting studies that aimed to increase healthy food purchases, found a statistically significant increase in purchase of target items and a statistically significant decrease in purchases of fat. Three studies aiming to increase purchases across a range of items only found differences for one of the products measured; Ball et al. (21) (targeting fruit, vegetables, and beverages) only detected a statistically significant increase in vegetable purchases but no differences in consumption; Geliebter et al. (29) (also targeting fruit, vegetables, and beverages) found an increase in purchasing of fruit and vegetables, but no differences in consumption and no differences in beverage purchases; and Budd et al. (23) only detected a statistically significant increase in healthier snack foods, despite also targeting beverages, vegetables, and whole-wheat bread. In Kristal et al. (32), a 50-cent coupon for fruit and vegetables affected neither purchase nor consumption. In contrast, in Waterlander et al. (47) a much larger, 50% discount led to a statistically significant increase in fruit and vegetable purchase and consumption. Ma et al. (34), reporting a study that aimed to decrease salt consumption through subsidy of a salt substitute, found a statistically significant increase in purchase of the salt substitute. In Phipps et al. (51), financial rewards postpurchase led to a statistically significant increase in fruit and vegetable purchase. Smith et al. (41) aimed to increase overall food purchase in food-insecure households and detected a statistically significant increase in food expenditure through provision of vouchers.

##### Intervention compared with intervention

In Brimblecombe et al. (22), both study arms received discounts on fruit, vegetables, and water, but one arm also included in-store posters, activity sheets, taste testing, and cooking demonstrations, and there was no control arm. The arm with the added components purchased significantly more vegetables, though no statistically significant differences in purchases were observed for the other target items. Dhar et al. (24), reporting a study that aimed to increase store profit, measured a statistically significant increase in 1 of the 2 target items with additional signage, over and above discounts alone (which also were associated with an increase).

#### Simulated experiments

Six studies tested economic interventions in simulated environments, and all detected a statistically significant effect on at least one of the measured outcomes. However, they varied in whether they impacted total energy purchased. In Epstein et al. (27), a study that aimed to increase nutrient quality and decrease energy, both subsidies (12.5% or 25% for healthier items) and taxes (12.5% or 25% for unhealthy items) led to an increase in purchases in the healthier items compared to control, but neither altered total energy purchased, whereas in Nederkoorn et al. (36), a 50% tax on high-energy foods significantly decreased total energy purchased compared with control. Waterlander et al. (45) used a factorial design to compare various discounts (none, 25% or 50% on healthier foods) and various taxes (5%, 10%, or 15% on unhealthier foods); participants receiving a 50% discount purchased significantly more healthy foods, but also purchased significantly more energy, which was the same in Waterlander et al. (46). There were no significant effects of the different price increases and no significant interactions. Waterlander et al. (44) compared a 25% discount on fruit and vegetables with no discount; the discount led to statistically significantly greater fruit and vegetable purchases with no differences in purchases in other food categories. In Waterlander et al. (48), a 19% tax on sugar-sweetened beverages led to significantly fewer purchases of these beverages.

### Group B: store environment interventions (no economic component)

Eleven studies, all in real stores, tested interventions altering the store environment that did not involve an economic component. None reported consumption as an outcome.

#### Intervention versus control

Eight studies in real stores compared a range of interventions with control, with mixed results. Of the 3 studies manipulating item availability (e.g., changing item stocking) among other components, 2 detected an effect. Dreze et al. (25) compared 2 different interventions with control: the intervention that involved changes to placement only (e.g., changes to where items are located in store, referred to by the authors as “space to movement”) resulted in changes opposite to the intended effect, whereas the intervention that also manipulated availability led to a statistically significant increase in 4 of the 5 categories targeted. In Ma et al. (34), stocking of a salt substitute and consumer education led to a statistically significant increase in salt substitute sales compared with control (no salt substitute). However, Lent et al. (33), which targeted students from grades 4 to 6 and involved a multicomponent intervention including item availability, other store changes, and other components delivered in school, failed to detect an effect for any of the outcomes measured.

The remaining 5 studies employed a range of different store environment changes without altering item availability. Two of the 5 detected a statistically significant effect: Foster et al. (28) used advertising, signage, item placement, and taste testing to promote healthier items in 5 product categories and detected a statistically significant increase for ≥1 product in favor of the intervention in each category, and Thorndike et al. (42) increased fruit and vegetable purchases through increasing visibility and quality of fresh produce. In contrast, in Jeffery et al. (31) signage, recipes, and brochures had no impact on the purchase of low-fat foods, and in Budd et al. (23) no significant difference was found for any of the outcomes measured when comparing an arm with advertising, signage, changes in shelf-height, taste testing, and consumer education with control. Finally, Russo et al. (40) tested 12 different types of signage; no significant differences were found when compared with each other or with a no-signage control.

#### Intervention compared with intervention

In Elofsson et al. (26), climate-related store signage increased sales of climate-certified milk compared with signage without climate-related information. In Milliron et al. (35), a nutrition-based intervention involving shelf-tags, educational leaflets, and an information session by an in-store dietitian led to statistically significant improvements in 2 of the 6 purchasing outcomes compared with shelf-tags alone. Finally, Wansink et al. (43) partitioned shopping carts, indicating a target proportion of the cart for produce, led to increased purchase fruits and vegetables, with the effect greatest when flyers highlighting nutritional benefits were distributed as opposed to flyers promoting cost savings.

### Group C: swaps

Three studies tested interventions that involved swaps, 2 in real environments and 1 in a simulated online grocery store. The 2 studies in real environments detected statistically significant effects: Huang et al. (30) automatically suggested swaps in an online supermarket and observed a statistically significant decrease in saturated fat purchased; and in Winnett et al. (50), a nutrition-based in-store computer kiosk in which participants entered their intended purchases and swaps were suggested to promote healthier choices led to statistically significant differences in favor of the intervention for 3 of the 7 target categories (the remaining 4 were not reported). In contrast, Forwood et al. (17) tested suggested swaps in a simulated online supermarket with a focus on reductions in energy density and did not detect any significant differences in purchasing.

### Group D: education/information only

Five studies tested interventions that involved only provision of consumer education/information or product labelling. All studies in this group included a no-intervention control. One measured consumption (21). Three studies tested the provision of consumer education in real stores, with mixed effects. In Achabal et al. (18), printed materials did not increase produce sales. In Ball et al. (21), newsletters and a supermarket tour did not change produce purchased, though self-reported vegetable consumption was statistically significantly higher in the intervention group. In Winnett et al. (49), an educational intervention primarily delivered in the home but including a supermarket visit with study staff reduced simple carbohydrate purchases, but did not alter the other 7 purchasing measures.

The remaining 2 studies evaluated nutritional labelling. In Ni Mhurchu et al. (38) there was no significant difference in healthiness of packaged food purchases when an app to show either traffic light labels or health star labels were compared with control in a physical supermarket. In an online experiment, Ducrot et al. (52) found that 3 of the 4 labels (5-color nutrition label, green tick, and multiple traffic lights) led to statistically significant benefits for ≥1 of the measured outcomes compared with control; labels based on guideline daily amounts did not show an effect.

### Differential effects by SES

Although many studies adjusted results by SES, only 6 presented analyses testing if results differed by SES (**Supplemental Table 4**). In 4 studies of in-store interventions, including labeling, in either real stores or simulated environments, there was some evidence of greater benefits for less-deprived groups. In the 2 studies of nutrition labels, Ni Mhurchu et al. (38) (real) found significant interactions by income with control more effective than traffic light or health star labels for low-income participants, and Ducrot et al. (16) (simulated) found that the effect of labels, though still present, was smaller in low-income participants. In the 2 studies of swaps in an online supermarket, Huang et al. (30) (real) found no significant difference in effect by education, employment, or income, whereas Forwood et al. (17) (simulated) found that less-deprived participants were more likely to accept swaps. However, in the 2 studies of price decreases that analyzed results by SES (1 real, 1 simulated), the intervention effects did not differ by income, education, or budget (37, 44).

### QCA

Results from our exploratory QCA are presented in **Supplemental Tables 5 and 6**. In summary, results pointed to the effectiveness of economic interventions regardless of setting and of environmental interventions and swap interventions in real-life settings (Supplemental Table 5). The 4 configurations associated with statistically significant changes in the desired direction for ≥1 of the foods targeted by the intervention were as follows: *1*) economic interventions in real and simulated grocery stores (without education, environmental components, or swaps); *2*) economic interventions in real grocery stores (without environmental components or swaps, and with/without education components; there were no studies in simulated environments which would have fitted this description); *3*) environmental interventions in real grocery stores (without swaps or economic or environmental components); and *4*) swaps with education in real grocery stores (without environmental or economic components). These configurations covered 85% of the effective interventions included in the QCA. When we tested the inverse—namely which configurations, if any, were not associated with statistically significant changes in the desired direction for at least one of the foods targeted by the intervention—the only intervention configuration that emerged was education in real grocery stores without economic components or swaps (Supplemental Table 6).

## DISCUSSION

This review includes 35 studies and, to the best of our knowledge, is the first to synthesize evidence from RCTs in grocery stores across a wide range of intervention types. The vast majority of studies (29 out of 35) aimed to improve health and we interpret the remainder in the context of their lessons for public health strategies. Overall, economic interventions showed the most promise, with 8 of the 9 studies in real store environments and all 6 studies in simulated environments detecting a statistically significant effect. The effects of these interventions appeared to be enhanced by additional promotional activity. Swap interventions appeared promising in real grocery stores, but only 2 studies tested them in this context. Interventions that altered the store environment showed mixed effects. In interventions that consisted solely of consumer education, findings were positive in simulated environments but for the most part no effect was detected in real grocery stores. The very limited data available suggested that the effects of economic interventions did not differ by SES, whereas studies of other in-store interventions presented evidence of both positive and negative impacts.

### Overall completeness and applicability of evidence

Although this review included 35 studies, with >20,000 participants across >800 grocery stores, important gaps in the evidence remain. All but one study was conducted in a high-income country, though interventions are also required in middle- and low-income countries, where grocery store shopping is on the rise and predicted increases in diet-related disease are the greatest (53, 54). In addition, due to the practical limitations of testing such interventions in RCTs, we found no interventions in real settings testing the effect of price increases. Only 5 studies measured consumption as well as purchasing, and these found mixed results. Research suggests that objectively documented household food purchases yield a reasonably accurate estimate of overall diet quality, but some caution must remain in interpreting purchasing as a proxy for consumption, particularly in regard to intake of specific nutrients (55). More studies are also needed to test whether the impact of interventions varies with SES, so as to avoid widening existing health disparities.

There are also questions about the applicability of this evidence. The interventions that appear most effective—namely, those manipulating price and those suggesting tailored swaps based on an individual's shopping list—may also be some of the most difficult to implement. Although some individual studies of environmental interventions showed promise, particularly regarding availability, these need replication before widespread implementation given mixed results across the body of evidence. This review also raises questions about the external validity of findings from simulated grocery stores, particularly with regard to educational interventions, which appear effective in simulated grocery stores and ineffective in real grocery stores. The lack of effect in real stores may be due to a greater lag between exposure to the message and enacting the behavior, or the presence of other competing information and cues that may influence purchasing decisions. Lastly, it is questionable whether findings from the 4 nonhealth-based interventions are directly translatable to public health interventions in a grocery store setting.

### Comparisons with other reviews in this area

Although other systematic reviews overlap in scope with ours, to the best of our knowledge this review is the most comprehensive and up-to-date in a field that has seen a recent upsurge in research. The most similar recent review to ours did not include pricing and product labeling and ran its searches in 2015; hence, it contains only 11 RCTs compared with our 35 (10). A 2013 review of grocery-store based interventions to promote health contains only 6 RCTs (9). In addition, we are the first review in this area to use QCA, a technique that augmented our narrative synthesis and was particularly valuable for exploring the variation in results between real and simulated settings.

Multiple reviews in this area have, as their headline conclusion, stated that more research is needed (9, 10, 56). Those reviews that drew conclusions on effectiveness are summarized below. Cameron et al. (10) noted that shelf-labeling interventions appeared promising when evaluating on a wide range of study types, but when restricted to RCTs, as in the present analysis, interventions of this type showed mixed results. The review by Adam et al. (11), which was limited to only obesity-related interventions, found that interventions combining price, information, and easy access and availability to health foods appeared promising but would need to be carefully implemented; findings on price and access were similar to ours. The review by Thow et al. (57), which evaluated taxes and subsidies only, was consistent with our review in concluding such interventions are likely to be effective in altering purchasing behavior.

### Strengths and limitations

This review uses gold standard methods, as set out by Cochrane, to minimize bias (13). Restricting to RCTs minimizes confounding, making us more confident in our results than some previous reviews, but arguably also restricts the nature of interventions that our review is able to evaluate. However, despite restricting our studies to RCTs, we judged 14 of the 35 studies to be at high risk of bias, and, despite searching trial databases and conference abstracts, cannot rule out the possibility of publication bias.

The scope of our review means that we are able to provide a broad appraisal of the evidence available for grocery store interventions across a range of settings, aims, and population groups, and to compare different intervention types. However, this inevitably brings with it heterogeneity and a large volume of data. When planning this review, we made a number of pragmatic decisions to deal with this heterogeneity. First, we chose to focus on results during or immediately postintervention and therefore are only able to draw conclusions regarding the effects of these interventions whilst implemented. Second, to synthesize the data we had to categorize it by intervention type; for some interventions (e.g., economic, education-only), this classification was relatively straightforward, but we are aware that other researchers may have defined some interventions differently, particularly labeling and swaps. In the absence of a clear consensus on how to group these types of interventions, we were guided by the existing literature. We also acknowledge that most studies were conducted in higher-income countries, limiting the generalizability to low- and middle-income countries, which may be the target of future studies aiming to improve diet as these countries undergo economic transition.

The explorative QCA represented a novel and empirically driven approach to help categorize and identify patterns in the studies we included. We recommend caution when interpreting the results of this analysis, as some studies measured the interventions’ impact on the purchase of multiple foods, thus inflating the probability of finding significant effects by chance.

Finally, our scope limits the amount of information we are able to present for each study. Our hope is that researchers and public health professionals aiming to explore more granular questions can use the data contained in this review, including the Supplementary data, as a starting point; interested readers are encouraged to contact the authors for further data.

### Conclusions

This review draws upon the best available evidence from RCTs and in doing so highlights the range of opportunities to change purchasing behaviors in grocery stores. Although the changes detected in purchasing were often small, given the scale of poor diet as a public health issue and the key role of grocery stores in shaping food consumption at a population level, our findings suggest interventions implemented in these settings—particularly ones that manipulate price, suggest swaps, and perhaps manipulate item availability—may play an important role in a multifaceted public health approach to reducing diet-related disease.

## Supplementary Material

Supplementary Data

## Notes

JH-B and SJ's time on this project, as well as project costs, were funded by the National Institute for Health Research (NIHR) Oxford Biomedical Research Centre (BRC) Obesity, Diet and Lifestyle Theme. FB's time on this project is funded by the Medical Research Council (MRC), the NIHR, and Green Templeton College, Oxford. SPR's time on this project is funded by the MRC and the British Heart Foundation (Clinical Research Training Fellowship). KF, RN and CP's time on this project was funded by the National Institute for Health Research (NIHR) Collaboration for Leadership in Applied Health Research and Care Oxford at Oxford Health NHS Foundation Trust. The views expressed are those of the author(s) and not necessarily those of the NHS, the NIHR, or the Department of Health. KF's time was also funded by Wolfson College, University of Oxford (Oxford Wolfson Marriott-Primary Care Graduate Scholarship). This research is part of the Wellcome Trust, Our Planet Our Health (Livestock, Environment and People - LEAP), award number 205212/Z/16/Z.

Supplemental Tables 1–6 and Supplemental Figure 1 are available from the “Supplementary data” link in the online posting of the article and from the same link in the online table of contents at https://academic.oup.com/ajcn/.

Abbreviations used: QCA, qualitative comparative analysis; RCT, randomized controlled trial; SES, socioeconomic status.
