# Supplementary material for: Grocery store interventions to change food purchasing behaviors: a systematic review of randomized controlled trials
Source: Am J Clin Nutr. 2018 Jun 4;107(6):1004–16. doi: 10.1093/ajcn/nqy045 (PMC5985731; doi:10.1093/ajcn/nqy045)
Supplement: Supplementary Data [file nqy045_supp.docx]

Contents

[Supplemental Table 1. MEDLINE search strategy 3](#_Toc504140040)

[Supplemental Figure 1. PRISMA diagram of study flow 4](#_Toc504140041)

[Supplemental Table 2. Further characteristics of included studies 5](#_Toc504140042)

[Supplemental Table 3. Further characteristics of included interventions 8](#_Toc504140043)

[Supplemental Table 4. Further purchasing and consumption data at our primary time point^,^ 12](#_Toc504140044)

[Achabal 1987 12](#_Toc504140045)

[Anderson 1997 12](#_Toc504140046)

[Ball 2015 12](#_Toc504140047)

[Ball 2016 13](#_Toc504140048)

[Brimblecombe 2017 13](#_Toc504140049)

[Budd 2017 14](#_Toc504140050)

[Dhar 1996 14](#_Toc504140051)

[Dreze 1994 15](#_Toc504140052)

[Ducrot 2016 16](#_Toc504140053)

[Elofsson 2016 17](#_Toc504140054)

[Epstein 2015 17](#_Toc504140055)

[Forwood 2015 17](#_Toc504140056)

[Foster 2014 17](#_Toc504140057)

[Geliebter 2013 18](#_Toc504140058)

[Huang 2006 18](#_Toc504140059)

[Jeffery 1982 18](#_Toc504140060)

[Kristal 1997 18](#_Toc504140061)

[Lent 2014 19](#_Toc504140062)

[Ma 2015 19](#_Toc504140063)

[Milliron 2012 19](#_Toc504140064)

[Nederkoorn 2012 19](#_Toc504140065)

[Ni Mhurchu 2010 20](#_Toc504140066)

[Ni Mhurchu 2017 20](#_Toc504140067)

[Phipps 2015 20](#_Toc504140068)

[Russo 1986 20](#_Toc504140069)

[Smith 2013 20](#_Toc504140070)

[Thorndike 2017 21](#_Toc504140071)

[Wansink 2017 21](#_Toc504140072)

[Waterlander 2012a 21](#_Toc504140073)

[Waterlander 2012b 22](#_Toc504140074)

[Waterlander 2013a 23](#_Toc504140075)

[Waterlander 2013b 23](#_Toc504140076)

[Waterlander 2014 24](#_Toc504140077)

[Winnett 1988 24](#_Toc504140078)

[Winnett 1991 24](#_Toc504140079)

[Supplemental Table 5. Results by socioeconomic status 25](#_Toc504140080)

[Supplemental Table 6. Results from QCA: combinations of study variables associated with statistically significant changes in purchasing in the desired direction for at least one of the foods targeted by the intervention 26](#_Toc504140081)

[Supplemental Table 7. Results from QCA: combination of study variables *not* associated with statistically significant changes in the desired direction in purchasing for at least one of the foods targeted by the intervention 27](#_Toc504140082)

[References to included studies 28](#_Toc504140083)

# Supplemental Table 1. MEDLINE search strategy

Run in MEDLINE (Ovid) 2 June 2017

| **#** | **Searches** |
| --- | --- |
| 1 | (Food Supply/ or *Food/) and Commerce/ |
| 2 | (supermarket? or superstore? or super store? or hypermarket?).ti,ab. |
| 3 | ((food or grocery) adj2 (store? or shop* or outlet? or market? or retailer?)).ti,ab. |
| 4 | (((small or medium or large or convenience or corner or local or community or neighbo?rhood?) adj2 (store? or shop*)) or newsagent?).ti,ab. |
| 5 | ((farm or farmer*) adj2 (store? or shop* or market?)).ti,ab. |
| 6 | (bakery or bakeries or butcher or butchers).ti,ab. |
| 7 | ((dollar or pound or discount) adj2 (store? or shop*)).ti,ab. |
| 8 | (((gas or gasoline or petrol) adj station?) or gasmart? or gas-mart?).ti,ab. |
| 9 | ((web* or internet or online or on-line or virtual* or 3d or 3-d or experimental or simulat* or laboratory or computer*) adj2 (store? or shop*)).ti,ab. |
| 10 | 1 or 2 or 3 or 4 or 5 or 6 or 7 or 8 or 9 |
| 11 | exp Food/ |
| 12 | beverages/ or exp carbonated beverages/ or energy drinks/ or "fruit and vegetable juices"/ |
| 13 | (grocer* or food* or fruit? or vegetable? or dairy or meat or fish or fat? or salt? or sugar? or sweet* or calori*).ti,ab. |
| 14 | (((soft or sugar? or sweet* or carbonated or energy or sport? or diet or flavo?red or fruit* or milk* or dairy or yoghurt or caffein* or cold or hot or nonalcohol* or non-alcohol*) adj3 (drink? or beverage?)) or soda? or flavo?red water? or fruit water? or cordial? or squash? or juice? or smoothie? or milkshake?).ti,ab. |
| 15 | ((drink? or beverage?) not (alcohol not (nonalcohol* or non-alcohol*))).ti. |
| 16 | (diet* or nutrition* or (health* adj2 eating)).ti,ab. |
| 17 | 11 or 12 or 13 or 14 or 15 or 16 |
| 18 | Choice Behavior/ or Consumer Behavior/ |
| 19 | Food Preferences/ |
| 20 | (purchas* or buy or buying or bought or sales or order*).ti,ab. |
| 21 | ((consumer? or shopping) adj2 (choice? or behavio*)).ti,ab. |
| 22 | (product? adj3 (select* or choice*)).ti,ab. |
| 23 | ((grocer* or food* or fruit? or vegetable? or dairy or meat or fish or fat? or salt? or sugar? or sweet* or calori*) adj3 (select* or choice*)).ti,ab. |
| 24 | 18 or 19 or 20 or 21 or 22 or 23 |
| 25 | 10 and 17 and 24 |
| 26 | randomized controlled trial.pt. |
| 27 | controlled clinical trial.pt. |
| 28 | randomized.ab. |
| 29 | randomly.ab. |
| 30 | trial.ab. |
| 31 | groups.ab. |
| 32 | 26 or 27 or 28 or 29 or 30 or 31 |
| 33 | exp animals/ not humans.sh. |
| 34 | 32 not 33 |
| 35 | 25 and 34 |

# Supplemental Figure 1. PRISMA diagram of study flow

Records excluded
(n = 1332)

Records screened
(n = 1467)

Records after duplicates removed
(n = 1467)

Identification

Eligibility

Included

Screening

Studies included in qualitative synthesis
(n = 35 )

Full-text studies assessed for eligibility
(n = 135)

Additional records identified through other sources
(n = 1)

Records identified through database searching
(n = 1619)

Full-text articles excluded, with reasons
(n = 100)

27 not RCT

24 did not measure purchasing

16 not grocery store

13 review

10 full text not available

5 ongoing/protocol only

5 other

# Supplemental Table 2. Further characteristics of included studies

| **Study ID** | **Mean age** | **weight** | **% F** | **Ethnicity** | **Mean household size** | **Area characteristics** | **Nature of outcome (objective or self-report)** |
| --- | --- | --- | --- | --- | --- | --- | --- |
| Achabal 1987(1) | NR^1^ | NR | NR | NR | NR | metropolitan | Purchase data: objective (weekly sales data) Consumption data: NA^2^ |
| Anderson 1997(2) | 52 | NR | 86 | Majority white | 3 | small towns | Purchase data: objective (annotated purchase receipts) Consumption data: NA |
| Ball 2015(3) | 43 | NR | 100 | Majority white | NR | NR | Purchase data: objective (transaction data) Consumption data: self-report (surveys) |
| Ball 2016(4) | 42 | NR | 100 | Majority white | 3 | suburbs | Purchase data: objective (transaction data) Consumption data: self-report (surveys) |
| Brimblecombe 2017(5) | NR | NR | NR | NR | NR | rural | Purchase data: objective (weekly store sales data)  Consumption data: NA |
| Budd 2017(6) | NR | NR | NR | NR | NR | metropolitan | Purchase data: self-report (store impact questionnaire asking for sales and stocking information)  Consumption data: NA |
| Dhar 1996(7) | NR | NR | NR | NR | NR | metropolitan | Purchase data: objective (weekly store-level scanner data)  Consumption data: NA |
| Dreze 1994(8) | NR | NR | NR | NR | NR | urban and suburban | Purchase data: objective (weekly sales data)  Consumption data: NA |
| Ducrot 2016(9) | NR | 70% BMI<25 | 81 | NR | NR | NR | Purchase data: objective (shopping cart data) Consumption data: NA |
| Elofsson 2016(10) | NR | NR | NR | NR | NR | mix | Purchase data: objective (Daily scanner data) Consumption data: NA |
| Epstein 2015(11) | 43 | mean BMI 27.5 | 100 | Majority white | 4 | NR | Purchase data: objective (receipts from online supermarket) Consumption data: NA |
| Forwood 2015(11) | 45 | approx 45% BMI normal range | 60 | NR | NR | NR | Purchase data: objective (purchase data online supermarket) Consumption data: NA |
| Foster 2014(12) | NR | NR | NR | Approx. 80% "minority" | NR | urban | Purchase data: objective (weekly sales data)  Consumption data: NA |
| Geliebter 2013(13) | 38 | mean BMI 30.2 | 70 | NR | NR | urban | Purchase data: objective (transaction data) Consumption data: self-report (24h dietary recall) |
| Huang 2006(12) | 40 | mean BMI 27 | 88 | Majority white | 3 | NR | Purchase data: objective (purchase data online supermarket)  Consumption data: NA |
| Jeffery 1982(13) | NR | NR | NR | NR | NR | urban and suburban | Purchase data: objective (weekly sales data)  Consumption data: NA |
| Kristal 1997(14) | NR | NR | 84 | NR | NR | rural | Purchase data: self-report (exit interviews) Consumption data: self-report (take-home survey) |
| Lent 2014(15) | 11 | majority in healthy BMI centile | 57 | approx. 42% black; 30% Hispanic/ latino | NR | urban | Purchase data: self-report (interview at store exit) Consumption data: NA |
| Ma 2015(16) | NR | NR | NR | NR | NR | rural | Purchase data: self-report (monthly telephone sales survey)  Consumption data: NA |
| Milliron 2012(15) | 43 | mean BMI 27.6 | 81 | Majority white | 4 | urban | Purchase data: objective (grocery receipts, photographs of purchases and fieldnotes)  Consumption data: NA |
| Nederkoorn 2011(5) | 41 | mean BMI 25.8 | 76 | NR | 3 | NR | Purchase data: objective (online supermarket purchase data)  Consumption data: NA |
| NiMhurchu 2010(4) | 44 | NR | 86 | Majority white | 2 | NR | Purchase data: objective (electronic scanner sales data)  Consumption data: |
| NiMhurchu 2017(17) | 33 | NR | 89 | Majority white | 3 | NR | Purchase data: objective (3 measures: scans of packaged foods using smartphone application, photographs of receipts, hard-copies of receipts)  Consumption data: NA |
| Phipps 2015(4) | 50 | NR | 81 | Majority African American | 4 | urban | Purchase data: objective (point-of-sale purchase data)  Consumption data: NA |
| Russo 1986(5) | NR | NR | NR | NR | NR | suburban | Purchase data: objective (sales records)  Consumption data: NA |
| Smith 2013(17) | 37 | NR | 95 | Majority white | 4 | NR | Purchase data: objective and self-report (copies of shopping receipts and self-report forms for un-receipted food purchases)  Consumption data: NA |
| Thorndike 2017(18) | NR | NR | 55 | Majority Hispanic/ latino | 4 | urban | Purchase data: objective and self-report (monthly sales data for vouchers, exit interviews for self-reported purchase of fruits/vegetables)  Consumption data: NA |
| Wansink 2017(18) | NR | NR | NR | NR | NR | urban | Purchase data: objective (recorded data from shopping receipt)  Consumption data: NA |
| Waterlander 2012(a)(19) | NR | NR | 80 | Majority white | 3 | NR | Purchase data: objective (online supermarket purchase data)  Consumption data: NA |
| Waterlander 2012(b)(20) | NR | NR | 85 | NR | 3 | NR | Purchase data: objective (online supermarket purchase data)  Consumption data: NA |
| Waterlander 2013(a)(21) | NR | NR | 85 | NR | 3 | NR | Purchase data: objective (online supermarket purchase data)  Consumption data: NA |
| Waterlander 2013(b)(22) | 51 | approx 50% BMI normal range | 97 | Majority white | 3 | NR | Purchase data: objective (supermarket cash receipts)  Consumption data: self-report (food-frequency questionnaire) |
| Waterlander 2014(23) | 29 | NR | 77 | NR | NR | NR | Purchase data: objective (online supermarket purchase data)  Consumption data: NA |
| Winett 1988(24) | 39 | NR | NR | NR | NR | NR | Purchase data: self-report (weekly food purchases form)  Consumption data: NA |
| Winett 1991(25) | 40 | NR | 80 | NR | NR | NR | Purchase data: objective (shopping receipts)  Consumption data: NA |
| ^1^ NR not reported  ^2^ NA not applicable | | | | | | | |

# Supplemental Table 3. Further characteristics of included interventions

| **Study ID** | **Intervention group^1^** | **Intervention length** | **Intervention aim(s)** | **Theoretical basis of intervention** | **Intervention components** | **Outside store element?** |
| --- | --- | --- | --- | --- | --- | --- |
| Achabal 1987 (22) | D (arms 1&2) | 4 weeks | promote health; educate consumers | NR^2^ | Arms 1&2: education/ info | No |
| Anderson 1997 (26) | A (arm 1) | 14 weeks | promote health; educate consumers | NR | price decrease, education/ info | No |
| Ball 2015 (27) | A (arms 1&2) | 3 months | promote health; educate consumers; increase sales of specific item(s) | Social cognitive theory, social ecological theory | Arm 2: education/ info Arm 1: as per 2 + price decrease | Yes (arm 2 only) |
| Ball 2016 (28) | D (arm 1) | 6 months | promote health; educate consumers; increase sales of specific item(s) | Social cognitive theory | education/ info | Yes |
| Brimblecombe 2017 (29) | A (arms 1&2) | 6 months | promote health; educate consumers; increase sales of specific item(s); decrease sales of specific item(s) | Social cognitive theory | Arm 2: price decrease, advertising, signage Arm 1: as per 2 + education/info, taste testing | No |
| Budd 2017 (30) | A (arms 1&3); B (arm 2) | 6 months | promote health; educate consumers; increase sales of specific item(s); decrease sales of specific item(s) | Social cognitive theory, social ecological model, economics law of demand | Arm 1: price decrease, item availability Arm 2: advertising, signage, produce refrigerators, education/info, taste testing Arm 3: price decrease, advertising, signage, produce refrigerators, education/ info, taste testing | No |
| Dhar 1996 (31) | A (arms 1&2) | 1 week | increase profit; increase sales of specific item(s) | NR | Arm 1: price decrease Arm 2: as per 1 + signage | No |
| Dreze 1994 (32) | B (arms 1&2) | 16 weeks | increase profit | NR | Arm 1: item availability, changes in shelf-height Arm 2: as per 1 + item placement | No |
| Ducrot 2016 (16) | D (arms 1-4) | 1 day | promote health; educate consumers | NR | Labelling (arms 1-4) | No |
| Elofsson 2016 (33) | B (arms 1&2) | 4 weeks | educate consumers; increase sales of specific item(s) | NR | Arm 2: Item availability Arm 1: as per 2 + education/ info | No |
| Epstein 2015 (34) | A (arms 1-4) | 5 days | promote health | NR | Arms 1&2: price increase Arms 3&4: price decrease | No |
| Forwood 2015 (35) | C (arms 1-4) | 1 day | promote health | NR | Swaps (arms 1-4) | No |
| Foster 2014 (36) | B (arm 1) | 6 months | promote health; increase sales of specific item(s) | framework of the “4 Ps” of marketing—price, promotion, product, and placement | Advertising, signage, item placement, taste testing | No |
| Geliebter 2013 (37) | A (arm 1) | 8 weeks | promote health; increase sales of specific item(s) | NR | Price decrease | No |
| Huang 2006 (38) | C (arm 1) | 5 months | promote health; educate consumers | NR | Swaps and education/info | No |
| Jeffery 1982 (39) | B (arm 1) | 6 months | promote health; educate consumers | NR | Signage and education/info | No |
| Kristal 1997 (40) | A (arm 1) | 8 months | promote health; educate consumers; increase sales of specific item(s); decrease sales of specific item(s) | Consumer Information Processing model | Price decrease, advertising, signage, education/ info, food demonstration | No |
| Lent 2014 (41) | B (arm 1) | 2 years | promote health; educate consumers; increase sales of specific item(s) | Social cognitive theory | Advertising, item availability, signage, education/ info, item placement, refrigerator units | Yes |
| Ma 2015 (42) | A (arm 1); B (arm 2) | 21 months | promote health; educate consumers; increase sales of specific item(s); decrease sales of specific item(s) | health belief model | Arm 1: price decrease, item availability, education/ info Arm 2: as per 1 but no price decrease | Yes (arms 1&2) |
| Milliron 2012 (43) | B (arms 1&2) | 4 months | promote health; educate consumers; increase sales of specific item(s); decrease sales of specific item(s) | NR | Item availability and education/ info (arms 1&2) | No |
| Nederkoorn 2011 (16) | A (arm 1) | 1 day | promote health; decrease sales of specific item(s) | NR | Price increase | No |
| NiMhurchu 2010 (21) | A (arm 1) | 6 months | promote health; increase sales of specific item(s) | NR | Arm 1: price decrease Arm 2: as per 1 + education/ info | Yes (arm 2 only) |
| NiMhurchu 2017 (44) | D (arms 1&2) | 4 weeks | promote health; educate consumers | NR | Labelling, education/ info (arms 1&2) | No |
| Phipps 2015 (45) | A (arm 1) | 8 weeks | promote health; increase sales of specific item(s) | NR | Financial rewards | No |
| Russo 1986 (46) | B (arms 1-12) | 12 weeks | promote health; educate consumers | NR | Item availability and education/ info (arms 1-12) | No |
| Smith 2013 (30) | A (arm 1) | 4 weeks | increase food purchasing | NR | Price decrease | No |
| Thorndike 2017 (47) | B (arm 1) | 5 months | promote health; increase sales of specific item(s) | NR | Signage and item placement | No |
| Wansink 2017 (48) | B (arms 1-6) | 1 day | promote health; educate consumers; increase sales of specific item(s) | Implied social norms | Partitioned grocery carts and education/ info (arms 1-6) | No |
| Waterlander 2012(a) (49) | A (arm 1) | 1 day | promote health; increase sales of specific item(s) | NR | Price decrease | No |
| Waterlander 2012(b) (50) | A (arm 1) | 1 day | promote health; increase sales of specific item(s); decrease sales of specific item(s) | NR | Arms 4-9: price decrease Arms 1-3: as per 4-9 + price increase | No |
| Waterlander 2013(a) (24) | A (arm 1) | 1 day | promote health; educate consumers; increase sales of specific item(s) | NR | Price decrease, signage, education/ info (arms 1-9) | No |
| Waterlander 2013(b) (25) | A (arm 1) | 6 months | promote health; educate consumers; increase sales of specific item(s) | Principles of motivational interviewing, theory of planned behaviour, self-regulatory action and coping planning (education arm) | Arm 1: price decrease Arm 2: as per arm 1 + education/ info | Yes (arm 2 only) |
| Waterlander 2014 (23) | A (arm 1) | 1 day | promote health; decrease sales of specific item(s) | NR | Price increase | No |
| Winett 1988 (51) | D (arm 1) | 7 weeks | promote health; educate consumers; increase sales of specific item(s); decrease sales of specific item(s); reduce cost of food purchases | Social cognitive theory | Education/ info | Yes |
| Winett 1991 (52) | C (arm 1) | 8 weeks | promote health; educate consumers; increase sales of specific item(s); decrease sales of specific item(s) | Social cognitive, health belief, and communication principles | Swaps + education/ info | No |
| ^1^ A: Economic interventions (any intervention including a price increase, decrease, or financial reward); B: Store environment changes (any intervention involving changes to the micro-environment, but not including economic interventions which are covered by (A), swaps which are covered by (C) or interventions based on product labelling or consumer education alone which are covered by (D)); C: Swap interventions, which offer consumers the opportunity to replace their usual food with a healthier alternative (but not including economic interventions which are covered by (A)); D: Labelling and/or educational interventions (interventions involving product labelling and/or consumer education/information, but not economic or other store environment changes)  ^2^ NR: not reported | | | | | | |

# Supplemental Table 4. Further purchasing and consumption data at our primary time point^[[1]](#footnote-2),^^[[2]](#footnote-3)^

## Achabal 1987

| Outcome - PURCHASES | Comparison | Between group difference | *P* value |
| --- | --- | --- | --- |
| Aggregate purchases over 6 target items | 3-way comparison (ANOVA) | Not reported (NR) | 0.505 |

## Anderson 1997

| Outcome - PURCHASES | Comparison | Between group difference (beta) | *P* value |
| --- | --- | --- | --- |
| Individual purchases of fat (g) | Intervention vs. control | -0.173 | <0.05 |
| Individual purchases of fibre (g) | Intervention vs. control | +0.184 | <0.01 |
| Individual purchases of fruit and vegetables (g) | Intervention vs. control | +0.198 | <0.01 |

## Ball 2015

| Outcome - PURCHASES | Comparison | Between group difference (beta (95% CI)) | *P* value |
| --- | --- | --- | --- |
| Total vegetable (g/week) | Intervention 1 (price reduction) vs. control | 232.7 (3.8, 461.6) | 0.046 |
|  | Intervention 2 (skill building & price reduction) vs. control | 137.6 (-90.3, 365.6) | 0.237 |
| Total fruit (g/week) | Intervention 1 (price reduction) vs. control | 363.9 (95.2, 632.5) | 0.008 |
|  | Intervention 2 (skill building & price reduction) vs. control | 279.9 (26.7, 533.1) | 0.030 |
| Water (mL/week) | Intervention 1 (price reduction) vs. control | 203.6 (-87.3, 494.5) | 0.170 |
|  | Intervention 2 (skill building & price reduction) vs. control | 31.2 (-255.9, 318.4) | 0.831 |
| Sugar sweetened beverages (mL/week) | Intervention 1 (price reduction) vs. control | 386.2 (-52.1, 824.5) | 0.084 |
|  | Intervention 2 (skill building & price reduction) vs. control | 881.4 (-686.6, 2449.5) | 0.271 |
| Diet beverages (mL/week) | Intervention 1 (price reduction) vs. control | 74.2 (-200.7, 349.0) | 0.597 |
|  | Intervention 2 (skill building & price reduction) vs. control | 381.5 (-277.5, 1040.5) | 0.257 |

| Outcome - CONSUMPTION | Comparison | Between group difference (beta (95% CI)) | *P* value |
| --- | --- | --- | --- |
| Total vegetable (g/week) | Intervention 1 (price reduction) vs. control | -25.8 (-145.4, 93.8) | 0.672 |
|  | Intervention 2 (skill building & price reduction) vs. control | 25.5 (-99.6, 150.6) | 0.689 |
| Total fruit (g/week) | Intervention 1 (price reduction) vs. control | 167.0 (-26.4, 360.4) | 0.091 |
|  | Intervention 2 (skill building & price reduction) vs. control | 157.9 (-45.0, 364.7) | 0.135 |
| Tap water (mL/week) | Intervention 1 (price reduction) vs. control | -198.6 (-629.9, 232.7) | 0.367 |
|  | Intervention 2 (skill building & price reduction) vs. control | 6.5 (-441.0, 454.0) | 0.977 |
| Bottled water (mL/week) | Intervention 1 (price reduction) vs. control | 34.0 (-299.0, 367.0) | 0.841 |
|  | Intervention 2 (skill building & price reduction) vs. control | 354.6 (-29.4, 738.5) | 0.070 |
| Sugar sweetened beverages (mL/week) | Intervention 1 (price reduction) vs. control | 73.4 (0.7, 146.2) | 0.048 |
|  | Intervention 2 (skill building & price reduction) vs. control | 30.0 (-17.8, 77.7) | 0.219 |
| Diet beverages (mL/week) | Intervention 1 (price reduction) vs. control | 46.3 (-47.3, 139.9) | 0.332 |
|  | Intervention 2 (skill building & price reduction) vs. control | 52.9 (-53.4, 159.3) | 0.329 |

## Ball 2016

| Outcome - PURCHASES | Comparison | Between group difference (beta (95% CI)) | *P* value |
| --- | --- | --- | --- |
| Total vegetable (g/week) | Intervention vs. control | 19.24 (-158.21, 196.70) | 0.832 |
| Total fruit (g/week) | Intervention vs. control | -10.06 (-176.65, 156.52) | 0.906 |

| Outcome - CONSUMPTION | Comparison | Between group difference (beta (95% CI)) | *P* value |
| --- | --- | --- | --- |
| Total vegetable (servings/day) | Intervention vs. control | 0.49 (0.25, 0.72) | <0.001 |
| Total fruit (servings/day) | Intervention vs. control | -0.05 (-0.30, 0.19) | 0.666 |

## Brimblecombe 2017

| Outcome - PURCHASES | Comparison | Between group difference (% change (95% CI)) | *P* value |
| --- | --- | --- | --- |
| Total fruit & vegetable (kg) | Discount + env. vs. discount alone | 7.6 % (-3.6, 20.2) | 0.19 |
| Total vegetable (kg) | Discount + env. vs. discount alone | 13.6 % (2.6, 25.7) | 0.014 |
| Total fruit (kg) | Discount + env. vs. discount alone | 2.1 % (-13.9, 21.0) | 0.81 |
| Water (kg) | Discount + env. vs. discount alone | 9.8 % (-11.9, 36.8) | 0.41 |
| Sugar sweetened beverages (kg) | Discount + env. vs. discount alone | 7.0 % (-5.8, 21.5) | 0.30 |
| Diet beverages (kg) | Discount + env. vs. discount alone | 2.7 % (-13.7, 22.4) | 0.76 |

## Budd 2017

| Outcome - PURCHASES | Comparison | Between group difference (beta(SE)) | *P* value |
| --- | --- | --- | --- |
| Sales (units) of all promoted foods | Intervention 1 (pricing) vs. control | 1.2 (31.8) | >0.05 |
|  | Intervention 2 (communication) vs. control | -8.1 (29.2) | >0.05 |
|  | Intervention 3 (both combined) vs. control | 11.8 (18.3) | >0.05 |
| Sales (units) of promoted beverages (phase 1) | Intervention 1 (pricing) vs. control | 3.3 (12.9) | >0.05 |
|  | Intervention 2 (communication) vs. control | -10.7 (29.3) | >0.05 |
|  | Intervention 3 (both combined) vs. control | 5.9 (5.0) | >0.05 |
| Sales (units) of promoted staple foods (phase 2) | Intervention 1 (pricing) vs. control | -5.6 (12.1) | >0.05 |
|  | Intervention 2 (communication) vs. control | -0.3 (2.1) | >0.05 |
|  | Intervention 3 (both combined) vs. control | -0.6 (4.2) | >0.05 |
| Sales (units) of promoted snack foods (phase 3) | Intervention 1 (pricing) vs. control | 3.6 (18.8) | >0.05 |
|  | Intervention 2 (communication) vs. control | 2.9 (8.0) | >0.05 |
|  | Intervention 3 (both combined) vs. control | 6.4 (13.9) | P<0.05 |

## Dhar 1996

| Outcome - PURCHASES | Comparison | Between group difference (beta) | *P* value |
| --- | --- | --- | --- |
| Sales of sugar sweetened beverages (units) | Intervention 1 (coupons) vs. Intervention 2 (bonus buys) | NR | <0.05 |
| Sales of ready to eat cereal (units) | Intervention 1 (coupons) vs. Intervention 2 (bonus buys) | NR | >0.05 |

## Dreze 1994

| Outcome - PURCHASES | Comparison | Between group difference (mean difference in % change) | *P* value |
| --- | --- | --- | --- |
| Sales of bottled juices ($ sales) | Intervention 1 (space to movement) vs. control | +4.9% | <0.001 |
|  | Intervention 2 (product reorganisation) vs.  control | NR | NR |
| Sales of canned soup ($ sales) | Intervention 1 (space to movement) vs. control | +6.3% | <0.001 |
|  | Intervention 2 (product reorganisation) vs.  control | -6% | <0.05 |
| Sales of canned seafood ($ sales) | Intervention 1 (space to movement) vs. control | -1% | 0.09 |
|  | Intervention 2 (product reorganisation) vs.  control | NR | NR |
| Sales of frozen entrees ($ sales) | Intervention 1 (space to movement) vs. control | +4.4% | <0.001 |
|  | Intervention 2 (product reorganisation) vs.  control | NR | NR |
| Sales of refrigerated juices ($ sales) | Intervention 1 (space to movement) vs. control | +2.6% | <0.001 |
|  | Intervention 2 (product reorganisation) vs.  control | NR | NR |
| Sales of cereals ($ sales) | Intervention 1 (space to movement) vs. control | NR | NR |
|  | Intervention 2 (product reorganisation) vs.  control | +5% | 0.08 |

## Ducrot 2016

| Outcome - PURCHASES | Comparison | Between group difference (beta) | *P* value |
| --- | --- | --- | --- |
| Overall nutritional quality (FSA/100g) | Intervention 1 (5 Colour Nutrition Label) vs. control | NR | <0.05 |
|  | Intervention 2 (Multiple traffic lights) vs. control | NR | <0.05 |
|  | Intervention 3 (Green ticks) vs. control | NR | <0.05 |
|  | Intervention 4 (Guideline Daily Amounts) vs. control | NR | NS |
| Total energy (kcal/100g) | Intervention 1 (5 Colour Nutrition Label) vs. control | NR | <0.05 |
|  | Intervention 2 (Multiple traffic lights) vs. control | NR | <0.05 |
|  | Intervention 3 (Green ticks) vs. control | NR | <0.05 |
|  | Intervention 4 (Guideline Daily Amounts) vs. control | NR | NS |
| Total fats (g/100g) | Intervention 1 (5 Colour Nutrition Label) vs. control | NR | <0.05 |
|  | Intervention 2 (Multiple traffic lights) vs. control | NR | NS |
|  | Intervention 3 (Green ticks) vs. control | NR | <0.05 |
|  | Intervention 4 (Guideline Daily Amounts) vs. control | NR | NS |
| Saturated fat (g/100g) | Intervention 1 (5 Colour Nutrition Label) vs. control | NR | <0.05 |
|  | Intervention 2 (Multiple traffic lights) vs. control | NR | NS |
|  | Intervention 3 (Green ticks) vs. control | NR | NS |
|  | Intervention 4 (Guideline Daily Amounts) vs. control | NR | NS |
| Sugars (g/100g) | Intervention 1 (5 Colour Nutrition Label) vs. control | NR | NS |
|  | Intervention 2 (Multiple traffic lights) vs. control | NR | NS |
|  | Intervention 3 (Green ticks) vs. control | NR | NS |
|  | Intervention 4 (Guideline Daily Amounts) vs. control | NR | NS |
| Total protein (g/100g) | Intervention 1 (5 Colour Nutrition Label) vs. control | NR | NS |
|  | Intervention 2 (Multiple traffic lights) vs. control | NR | NS |
|  | Intervention 3 (Green ticks) vs. control | NR | NS |
|  | Intervention 4 (Guideline Daily Amounts) vs. control | NR | NS |
| Fibre (g/100g) | Intervention 1 (5 Colour Nutrition Label) vs. control | NR | NS |
|  | Intervention 2 (Multiple traffic lights) vs. control | NR | <0.05 |
|  | Intervention 3 (Green ticks) vs. control | NR | NS |
|  | Intervention 4 (Guideline Daily Amounts) vs. control | NR | NS |
| Sodium (mg/100g) | Intervention 1 (5 Colour Nutrition Label) vs. control | NR | <0.05 |
|  | Intervention 2 (Multiple traffic lights) vs. control | NR | NS |
|  | Intervention 3 (Green ticks) vs. control | NR | NS |
|  | Intervention 4 (Guideline Daily Amounts) vs. control | NR | NS |

## Elofsson 2016

| Outcome - PURCHASES | Comparison | Between group difference (% difference (SE)) | *P* value |
| --- | --- | --- | --- |
| Sales of climate certified milk (log)* | Intervention vs. control | +6.33% (0.029) | <0.05 |

*Climate-certified according to the Swedish standards for Climate Certification of Food (CCF). The CCF is a voluntary labelling scheme that requires certified food producers to strive towards a significant reduction of GHG emissions by focusing on the production choices with the largest climate impact(48).

There is also an estimate which doesn’t control for daily turnover (+5%, p=0.125), the one above controlled for store/week as well as daily turnover as fixed effects

## Epstein 2015

| Outcome - PURCHASES | Comparison* | Between group difference (coefficient (95% CI)) | *P* value |
| --- | --- | --- | --- |
| Total energy (kcal) from subsidized food | Subsidies vs. control | 13.74 (8.51, 18.97) | <0.001 |
|  | Taxes vs. control | -6.61 (-11.94,  -1.28) | 0.02 |
| Total energy (kcal) | Subsidies vs. control | -14.37 (-33.54,  4.81) | 0.14 |
|  | Taxes vs. control | -17.68 (-37.03,  1.68) | 0.07 |

*Factorial trial, individual intervention vs. control comparisons not reported

## Forwood 2015

| Outcome - PURCHASES | Comparison | Between group difference (mean difference (95% CI)) | *P* value |
| --- | --- | --- | --- |
| Total basket energy density (kJ/100g) | All interventions vs. control | -24.1 (4.04,  -52.23) | NS |
|  | Intervention 1 (consented swaps) vs. Intervention 2 (imposed swaps) | 22.8 (-3.69, 49.26) | NS |
|  | Intervention 3 (at selection) vs. Intervention 4 (at check out) | 3.53 (-22.94, 20.01) | NS |
| FSA nutrient profile of treat item | All interventions vs. control | 0.62 (-1.87, 3.11) | 0.626 |
|  | Intervention 1 (consented swaps) vs. Intervention 2 (imposed swaps) | 0.155 (-2.67, 2.98) | 0.915 |
|  | Intervention 3 (at selection) vs. Intervention 4 (at check out) | -1.55 (−4.38, 0.13) | 0.282 |

## Foster 2014

| Outcome - PURCHASES | Comparison | Between group difference (mean difference in change pre/post (SE)) | *P* value |
| --- | --- | --- | --- |
| Whole milk (oz) | Intervention vs. control | -3910.6 (4942.7) | 0.6658 |
| 2% milk (oz) | Intervention vs. control | -2417.9 (3410.9) | 0.5505 |
| 1% milk (oz) | Intervention vs. control | 3383.2 (1403.8) | 0.0014 |
| Skimmed milk (oz) | Intervention vs. control | 1509.1 (1079.9) | 0.0078 |
| Cheerios (oz) | Intervention vs. control | 162.6 (187.4) | 0.3905 |
| Honeycomb cereal (oz) | Intervention vs. control | 41.0 (55.9) | 0.2271 |
| Frozen steak (units) | Intervention vs. control | 10.5 (12.1) | 0.6503 |
| Frozen chicken nuggets (units) | Intervention vs. control | 20.5 (10.4) | 0.0074 |
| Frozen turkey dinner (units) | Intervention vs. control | 10.8 (6.2) | 0.0326 |
| In aisle Pepsi (oz) | Intervention vs. control | -2706.5 (6250.6) | 0.8793 |
| In aisle diet Pepsi (oz) | Intervention vs. control | -507.0 (970.5) | 0.2905 |
| In aisle water (oz) | Intervention vs. control | 1690.0 (6649.8) | 0.0109 |
| Checkout sugar sweetened beverages (units) | Intervention vs. control | -13.5 (9.3) | 0.6173 |
| Checkout diet beverages (units) | Intervention vs. control | 1.5 (4.4) | 0.2219 |
| Checkout water (units) | Intervention vs. control | 18.5 (6.0) | 0.0002 |

## Geliebter 2013

| Outcome - PURCHASES | Comparison | Between group difference (beta) | *P* value |
| --- | --- | --- | --- |
| Weekly purchases of fruit & vegetables (unit not specified) | Intervention vs. control | NR | <0.001 |
| Weekly purchases of diet beverages (unit not specified) | Intervention vs. control | NR | NR |

| Outcome - CONSUMPTION | Comparison | Between group difference (beta) | *P* value |
| --- | --- | --- | --- |
| Intake of fruit & vegetables (g/day) | Intervention vs. control | NR | NS* |
| Intake of diet beverages (g/day | Intervention vs. control | NR | NS |
| Intake of sugar sweetened beverages (kcal/day) | Intervention vs. control | NR | NS |
| Total energy intake (kcal/day) | Intervention vs. control | NR | NS |

*between group difference not reported but authors state in abstract “intake of F&V increased from baseline to intervention in the discount group relative to the control group (p=0.037)”. However, this result not reported in main text, where authors only state “group-by-time interaction approached significance (p=0.067)”

## Huang 2006

| Outcome - PURCHASES | Comparison | Between group difference (beta (95% CI)) | *P* value |
| --- | --- | --- | --- |
| Saturated fat (% from energy) | Intervention vs. control | -0.66 (0.48, 0.84) | <0.001 |

## Jeffery 1982

| Outcome - PURCHASES | Comparison | Between group difference (difference in changes from baseline) | *P* value |
| --- | --- | --- | --- |
| Weekly average sales of low fat frozen desserts | Intervention vs. control | Increased | NS |
| Weekly average sales of low fat cottage cheese | Intervention vs. control | Increased | NS |

## Kristal 1997

| Outcome - PURCHASES | Comparison | Between group difference | *P* value |
| --- | --- | --- | --- |
| Fruit & Vegetable purchases at 1 year (percentage purchasing fruit or vegetable on day interviewed) | Intervention vs. control | NR. At baseline, 71.6% intervention and 70.4% control. At 1 year, 80.3% intervention and 78.7% control. | >0.05 |

| Outcome - CONSUMPTION | Comparison | Between group difference (beta) | *P* value |
| --- | --- | --- | --- |
| Fruit & Vegetable intake at 1 year (servings/day) | Intervention vs. control | NR. At baseline, mean 3.21 (SD 1.75) intervention, 3.14 (SD 1.74) control. At 1 year, mean 3.54 (1.79) intervention, 3.44 (1.83) control. | >0.05 |

## Lent 2014

| Outcome - PURCHASES | Comparison | Between group difference (odds ratio (95%CI)) | *P* value |
| --- | --- | --- | --- |
| Energy (kcal) | Control vs. intervention | 0.88 (0.5, -1.5) | 0.58 |
| Fat (g) | Control vs. intervention | 0.77 (0.5, -1.3) | 0.32 |
| Sodium (mg) | Control vs. intervention | 1.21 (0.7, -2.2) | 0.53 |
| Carbohydrates (g) | Control vs. intervention | 1.21 (0.7, -2.1) | 0.50 |
| Sugars (g) | Control vs. intervention | 0.84 (0.4, -1.6) | 0.61 |
| Protein (g) | Control vs. intervention | 1.17 (0.7, -2.1) | 0.60 |
| Fibre (g) | Control vs. intervention | 0.78 (0.5, -1.5) | 0.45 |

## Ma 2015

| Outcome - PURCHASES | Comparison | Between group difference (sales in intervention vs. control (95% CI)) | *P* value |
| --- | --- | --- | --- |
| Monthly sales of salt (kg) | Intervention 1 (price subsidy & health education) vs. control | 35.80 (21.54, 50.06) | <0.001 |
|  | Intervention 2 (health education) vs. control | 16.99 (2.66, 31.33) | 0.020 |

## Milliron 2012

| Outcome - PURCHASES | Comparison | Between group difference (beta) | *P* value |
| --- | --- | --- | --- |
| Total fat (g/1000 kcal) | Intervention vs. control | NR | 0.494 |
| Saturated fat (g/1000 kcal) | Intervention vs. control | NR | 0.460 |
| Trans fat (g/1000 kcal) | Intervention vs. control | NR | 0.993 |
| Fruit servings (g/1000 kcal) | Intervention vs. control | NR | 0.002 |
| Vegetable servings (g/1000 kcal) | Intervention vs. control | NR | 0.063 |
| Dark green/yellow vegetables (servings/1000 kcal) | Intervention vs. control | NR | 0.034 |

## Nederkoorn 2012

| Outcome - PURCHASES | Comparison | Between group difference (ΔR2) | *P* value |
| --- | --- | --- | --- |
| Total energy (kcal) | Intervention vs. control | 0.021 | <0.01 |
| Energy from low energy density foods | Intervention vs. control | NR | NR |
| Energy from high energy density foods | Intervention vs. control | 0.026 | <0.01 |
| % Energy from high energy density foods | Intervention vs. control | 0.018 | <0.01 |
| Total energy per person (kcal) | Intervention vs. control | NR | NR |

## Ni Mhurchu 2010

| Outcome - PURCHASES | Comparison | Between group difference (beta (95% CI)) | *P* value |
| --- | --- | --- | --- |
| Saturated fat (% from total energy) | Intervention vs control | -0.02% (-0.40, 0.36) | 0.91 |
| Predefined healthier foods (kg/week) | Intervention vs control | 0.79 (0.43, 1.16) | <0.001 |

## Ni Mhurchu 2017

| Outcome - PURCHASES | Comparison | Between group difference  (mean difference (95% CI)) | *P* value |
| --- | --- | --- | --- |
| All foods (nutrient profile score) | Intervention 1 (Traffic light labels) vs. control | 0.08 (−0.38, 0.54) | 0.74 |
|  | Intervention 2 (Health Star Rating) vs. control | -0.22 (−0.68, 0.25) | 0.36 |
| All foods (transformed nutrient profile score) | Intervention 1 (Traffic light labels) vs. control | -0.20 (−0.94, 0.54) | 0.60 |
|  | Intervention 2 (Health Star Rating) vs. control | -0.60 (−1.35, 0.15) | 0.12 |
| All foods (weighed nutrient profile score) | Intervention 1 (Traffic light labels) vs. control | 0.13 (−0.83, 1.10) | 0.78 |
|  | Intervention 2 (Health Star Rating) vs. control | -0.22 (−1.19, 0.76) | 0.66 |

## Phipps 2015

| Outcome - PURCHASES | Comparison | Between group difference (beta (95% CI)) | *P* value |
| --- | --- | --- | --- |
| Fruit & Vegetables (servings/week) | Intervention vs. control | 10.2 (3.6, 25.7) | <0.001 |
| Vegetables (servings/week) | Intervention vs. control | 8 (1.5, 16.9) | <0.001 |
| Fruit (servings/week) | Intervention vs. control | 2.5 (0.3, 9.5) | 0.01 |

*Fully adjusted model adjusted for changes in prices

## Russo 1986

| Outcome - PURCHASES | Comparison | Between group difference (standard deviations below the mean) | *P* value |
| --- | --- | --- | --- |
| Overall nutritional quality (across all product categories) | Intervention vs. control | -0.029 | NS |
| Nutritional quality of individual product categories | Intervention vs. control | NR | NS |
| Other nutrition criteria (NS) | Intervention vs. control | NR | NS |
| Energy (kcal) | Intervention vs. control | NR | NS |
| Nutrients (NS) | Intervention vs. control | NR | NS |

## Smith 2013

| Outcome - PURCHASES | Comparison | Between group difference (adjusted (95% CI)) | *P* value |
| --- | --- | --- | --- |
| Total food expenditure ($NZ) | Intervention vs. control | 15.20 (1.46, 28.94) | 0.030 |
| Fruit & Vegetable expenditure ($NZ) | Intervention vs. control | 0.46 (-1.97, 2.89) | 0.709 |
| Meat & Poultry expenditure ($NZ) | Intervention vs. control | 0.29 (-3.07, 3.64) | 0.866 |
| Dairy expenditure ($NZ) | Intervention vs. control | 0.83 (-0.75, 2.42) | 0.302 |
| Wholegrain expenditure ($NZ) | Intervention vs. control | NR | 0.535 |

## Thorndike 2017

| Outcome - PURCHASES | Comparison | Between group difference (beta) | *P* value |
| --- | --- | --- | --- |
| Store sales of WIC fruit & vegetables | Intervention vs. control | 15.20 | 0.030 |

## Wansink 2017

| Outcome - PURCHASES | Comparison* | Between group difference (F) | *P* value |
| --- | --- | --- | --- |
| Fruit & Vegetable expenditure ($) | Comparing: control cart, 35% partition cart, and 50% partition cart ($10.36, $11.85, $13.40) | 10.15 | <0.01 |
|  | Comparing: Health/ Nutrition flyer compared to Value/Cost-Savings flyer ($14.42 vs. $9.18) | 72.66 | <0.01 |
|  | Comparing: in Health/Nutrition conditions, control cart, the 35% Partition cart, and the 50% Partition cart ($11.61, $14.97, and $17.54) | NR | <0.05 |
|  | Comparing: in Value/Cost-Savings conditions, control cart, the 35% Partition cart, and the 50% Partition cart ($8.77, $8.72, and $9.92) | NR | >0.05 |
| Meat & Treats expenditure ($) | Comparisons based on flyer, cart partitioning, or interactions between the two (Arm 1 $17.51; Arm 2 $14.60; Arm 3 $17.13; Arm 4 $16.09; Arm 5 $16.63; Arm 6 $16.35) | NR | NS |

*Factorial trial; individual intervention vs. control comparisons not presented in study report.

## Waterlander 2012a

| Outcome - PURCHASES | Comparison | Between group difference (beta (95% CI)) | *P* value |
| --- | --- | --- | --- |
| Total fruit & vegetables (number of items) | Intervention vs. control | 0.9 | 0.19 |
| Total fruit & vegetables (g) | Intervention vs. control | 605.0 | 0.16 |
| Total energy (kcal) | Intervention vs. control | -976 | 0.78 |
| Total fruit & vegetables (number of items) | Intervention vs. control* | 1.33 (-0.16, 2.82) | 0.08 |
| Total fruit & vegetables (g) | Intervention vs. control* | 984.0 (97, 1872) | 0.03 |
| Total energy (kcal) | Intervention vs. control* | 2,327 (-3494, 8147) | 0.43 |

*Adjusted models

## Waterlander 2012b

| Outcome - PURCHASES | Comparison | Between group difference (beta (95% CI)) | *P* value |
| --- | --- | --- | --- |
| Unhealthy foods (number) | Intervention 1 (50% discount) vs. control | 3.78 (-0.12, 7.68) | 0.06 |
|  | Intervention 2 (25% discount) vs. control | 1.75 (-2.06, 5.55) | NS |
|  | Intervention 3 (25% increased price) vs. control (5% increased price) | -1.99 (-5.82, 1.85) | NS |
|  | Intervention 4 (10% increased price) vs. control (5% increased price) | -1.66 (-5.47, 2.15) | NS |
| Healthy foods (number) | Intervention 1 (50% discount) vs. control | 6.62 (2.47, 10.78) | <0.01 |
|  | Intervention 2 (25% discount) vs. control | 1.75 (-2.30, 5.80) | NS |
|  | Intervention 3 (25% increased price) vs. control (5% increased price) | 1.03 (-3.05, 5.11) | NS |
|  | Intervention 4 (10% increased price) vs. control (5% increased price) | 0.48 (-3.58, 4.54) | NS |
| Total energy (kcal) | Intervention 1 (50% discount) vs. control | 10505 (4376, 16.635) | <0.01 |
|  | Intervention 2 (25% discount) vs. control | 4669 (-1305, 10.642) | NS |
|  | Intervention 3 (25% increased price) vs. control (5% increased price) | 816 (-5209, 6841) | NS |
|  | Intervention 4 (10% increased price) vs. control (5% increased price) | 313 (-5676, 6302) | NS |
| Vegetables (g) | Intervention 1 (50% discount) vs. control | 821(85.1, 1556) | <0.05 |
|  | Intervention 2 (25% discount) vs. control | 52 (-665, 769) | NS |
|  | Intervention 3 (25% increased price) vs. control (5% increased price) | 368 (-355, 1091) | NS |
|  | Intervention 4 (10% increased price) vs. control (5% increased price) | 121(-598, 840) | NS |
| Fruit (g) | Intervention 1 (50% discount) vs. control | 420 (-322, 1163) | NS |
|  | Intervention 2 (25% discount) vs. control | -382 (-1105, 341) | NS |
|  | Intervention 3 (25% increased price) vs. control (5% increased price) | 83.2 (646, 813) | NS |
|  | Intervention 4 (10% increased price) vs. control (5% increased price) | 304 (-421, 1029) | NS |

## Waterlander 2013a

| Outcome - PURCHASES | Comparison | Between group difference (beta (95% CI)) | *P* value |
| --- | --- | --- | --- |
| Healthy foods (number) | Intervention 1 (10% discount) vs. Intervention 2 (50% discount) | −8.58 (-13.4,  -3.75) | <0.01 |
|  | Intervention 3 (25% discount) vs. Intervention 2 (50% discount) | −9.02 (-14.0,  -4.05) | <0.01 |
|  | Intervention 1 (10% discount) vs. Intervention 3 (25% discount) | −0.44 (-5.57, 4.70) | NS |
| Healthy foods (%) | Intervention 1 (10% discount) vs. Intervention 2 (50% discount) | −4.02 (-10.3, 2.30) | NS |
|  | Intervention 3 (25% discount) vs. Intervention 2 (50% discount) | −6.55(-13.1,  -0.06) | NS |
|  | Intervention 1 (10% discount) vs. Intervention 3 (25% discount) | −2.53 (-9.24, 4.18) | NS |
| Unhealthy foods (number) | Intervention 1 (10% discount) vs. Intervention 2 (50% discount) | −3.20 (-8.42, 2.02) | NS |
|  | Intervention 3 (25% discount) vs. Intervention 2 (50% discount) | −1.39 (-6.76, 3.98) | NS |
|  | Intervention 1 (10% discount) vs. Intervention 3 (25% discount) | 1.81 (-3.74, 7.35) | NS |

## Waterlander 2013b

| Outcome - PURCHASES | Comparison | Between group difference (beta (95% CI)) | *P* value |
| --- | --- | --- | --- |
| Fruit & Vegetables (kg) | Intervention 1 (discount) vs. control | 5252 (2836, 7668) | <0.001 |
|  | Intervention 2 (education) vs. control | 34.7 (-2571, 2640) | NS |
|  | Intervention 3 (education & discount) vs. control | 5383 (2958, 7808) | <0.001 |
| Fruit & Vegetables (kg)* | Intervention 1 (discount) vs. control | 3894 (equivalent to +3.9 kg; 1500, 6287) | <0.01 |
|  | Intervention 2 (education) vs. control | 1075 (-1468, 3617) | NS |
|  | Intervention 3 (education & discount) vs. control | 5556 (equivalent to +5.6 kg; 3188, 7925) | <0.001 |

*Adjusted

| Outcome - CONSUMPTION | Comparison | Between group difference | *P* value |
| --- | --- | --- | --- |
| Percentage of participants who consumed sufficient (at least 400 g/day) amount of F&Vs | Intervention vs. control | NR* | NR |

*between group difference not reported. Authors state: “The percentage of participants who consumed sufficient amounts of F&Vs increased significantly from 42.5% at baseline to 61.3% at 6 months in the discount groups (P = 0.03). For the nondiscount groups, these percentages were 52.7% and 52.5%, respectively (P = 0.80).”

##

## Waterlander 2014

| Outcome - PURCHASES | Comparison | Between group difference (beta (95% CI)) | *P* value |
| --- | --- | --- | --- |
| Sugar sweetened beverages (l) | Intervention vs. control | NR | 0.09 |
| Sugar sweetened beverages (l)* | Intervention vs. control | -0.90 (−1.70, −.10) | <0.05 |

*Adjusted

## Winnett 1988

| Outcome - PURCHASES | Comparison | Between group difference (F) | *P* value |
| --- | --- | --- | --- |
| Complex carbohydrates (% energy) | Intervention vs. control | NR (% change intervention +1.5, control -1.2) | NR |
| Simple carbohydrates (% energy) | Intervention vs. control | 2.09 (% change intervention +5.1, control -0.0) | <0.05 |
| Total carbohydrates (% energy) | Intervention vs. control | NR (% change intervention +6.6, control -0.0) | NR |
| Protein (% energy) | Intervention vs. control | NR (% change intervention -1.7, control -1.6) | NR |
| Saturated fat (% energy) | Intervention vs. control | NR (% change intervention -2.2, control +1.3) | NR |
| Total fat (% energy) | Intervention vs. control | 2.13 (% change intervention -5.5, control 1.3) | P<0.005 |
| Food expenditure ($) | Intervention vs. control | NR (% change intervention -12.0, control -8.7) | NR |

## Winnett 1991

| Outcome - PURCHASES | Comparison | Between group difference (F) | *P* value |
| --- | --- | --- | --- |
| High fat meat (units) | Intervention vs. control | 14.87 (equates to approx. 37% decrease) | <0.001 |
| High fibre grains/cereals (units) | Intervention vs. control | 11.95 (equates to approx. 62% increase) | <0.001 |
| High fat dairy (units) | Intervention vs. control | 4.53 (equates to approx. 20% decrease) | <0.05 |
| Low fat dairy (units) | Intervention vs. control | NR | NR |
| Low fat meat (units) | Intervention vs. control | NR | NR |
| Fruit & Vegetables (units) | Intervention vs. control | NR | NR |
| Low fat fish & poultry (units) | Intervention vs. control | NR | NR |

# Supplemental Table 5. Results by socioeconomic status

| **Study ID** | **Intervention group^1^** | **Effects by SES** |
| --- | --- | --- |
| Ducrot 2016 (48) | D (arms 1-4) | Significant interaction between education status and intervention (p=0.004) and monthly income per household unit and intervention (p=0.0038), with smaller effect in individuals with lower income. |
| Forwood 2015 (37) | C (arms 1-4) | Collapsing by intervention group, female participants and less deprived participants (as indicated by higher IMD quintiles) were more likely to accept swaps. There was no effect of education on the acceptance of swaps. |
| Huang 2006 (23) | C (arm 1) | Authors state: “the intervention was similarly effective in most of the subgroups studied.” No significant difference in effect of intervention by university education (p=0.21), employment (p=0.64), or income (p=0.53). |
| NiMhurchu 2010 (44) | A (arm 1) | Association of price discounts with healthy food purchasing did not vary by household income or education. Authors state: “Beyond price discount effects by ethnicity in the first 6 months, there was no clear or meaningful patterns in any of the other interactions (income and education).” |
| NiMhurchu 2017 (28) | D (arms 1&2) | Significant interactions by income: nutrition information panels (control) more effective than health star ratings or traffic light labels for low-income participants (p=0.006). Authors state: “These differences may have occurred by chance because of the number of interactions that were tested and the very small number of participants in the pertinent subgroups.” No evidence that effects varied by education. |
| Waterlander 2012(a) (24) | A (arm 1) | No statistically significant interactions by shopping budget. |
| ^1^ A: Economic interventions (any intervention including a price increase, decrease, or financial reward); B: Store environment changes (any intervention involving changes to the micro-environment, but not including economic interventions which are covered by (A), swaps which are covered by (C) or interventions based on product labelling or consumer education alone which are covered by (D)); C: Swap interventions, which offer consumers the opportunity to replace their usual food with a healthier alternative (but not including economic interventions which are covered by (A)); D: Labelling and/or educational interventions (interventions involving product labelling and/or consumer education/information, but not economic or other store environment changes) | | |

# Supplemental Table 6. Results from QCA: combinations of study variables associated with statistically significant changes in purchasing in the desired direction for at least one of the foods targeted by the intervention **^[[3]](#footnote-4)^**

| **Configuration** | | **Raw coverage** | **Unique coverage** | **Consistency** |
| --- | --- | --- | --- | --- |
| **Definition** | **Boolean minimisation** |  |  |  |
| Economic only interventions in real and simulated environments | ~environ*~educ*Economic*~swap | 0.5 | 0.269231 | 0.928571 |
| Economic interventions without environmental components or swaps in real environments (with or without educational components) | ~environ*Economic*Real*~swap | 0.384615 | 0.153846 | 0.909091 |
| Environment only interventions in real environments | environ*~educ*~Economic*Real*~swap | 0.115385 | 0.115385 | 0.75 |
| Swap and education interventions in real environments without environmental or economic components | ~environ*educ*~Economic*Real*swap | 0.0769231 | 0.0769231 | 1 |

Solution coverage: 0.846154^[[4]](#footnote-5)^; Solution consistency: 0.916667^[[5]](#footnote-6)^

#

# Supplemental Table 7. Results from QCA: combination of study variables *not* associated with statistically significant changes in the desired direction in purchasing for at least one of the foods targeted by the intervention

| **Configuration** | | **Raw coverage** | **Unique coverage** | **Consistency** |
| --- | --- | --- | --- | --- |
| **Definition** | **Boolean minimisation** |  |  |  |
| Education only interventions in real environments | educ*~Economic*Real*~swap | 0.869565 | 0.869565 | 0.909091 |

Solution coverage: 0.869565^[[6]](#footnote-7)^; Solution consistency: 0.909091^[[7]](#footnote-8)^

# References to included studies

1. Abajobir AA, Abate KH, Abbafati C, Abbas KM, Abd-Allah F, Abdulle AM, Abera SF, Aboyans V, Abu-Raddad LJ, Abu-Rmeileh NME, et al. Global, regional, and national comparative risk assessment of 84 behavioural, environmental and occupational, and metabolic risks or clusters of risks, 1990&#x2013;2016: a systematic analysis for the Global Burden of Disease Study 2016. The Lancet;390(10100):1345-422. doi: 10.1016/S0140-6736(17)32366-8.

2. World Health Organization. Internet: <http://www.who.int/mediacentre/factsheets/fs311/en/> (accessed 30 Sept 2016.

3. Drewnowski A, Rehm CD. Energy intakes of US children and adults by food purchase location and by specific food source. Nutrition Journal 2013;12(1):59. doi: 10.1186/1475-2891-12-59.

4. Shemilt I, Hollands GJ, Marteau TM, Jebb SA, Kelly MP, Nakamura R, Suhrcke M, Ogilvie D. Effects of changes in the economic environment on diet-and physical activity related behaviours and corollary outcomes: a large-scale scoping review. 2013.

5. Hollands GJ, Shemilt I, Marteau TM, Jebb SA, Kelly MP, Nakamura R, Suhrcke M, Ogilvie D. Altering micro-environments to change population health behaviour: towards an evidence base for choice architecture interventions. BMC Public Health 2013;13(1):1218. doi: 10.1186/1471-2458-13-1218.

6. Marteau TM, Hall PA. Breadlines, brains, and behaviour. BMJ 2013;347:f6750.

7. Dinsa GD, Goryakin Y, Fumagalli E, Suhrcke M. Obesity and socioeconomic status in developing countries: a systematic review. Obesity Reviews 2012;13(11):1067-79. doi: 10.1111/j.1467-789X.2012.01017.x.

8. Ball K, Crawford D. Socioeconomic status and weight change in adults: a review. Social Science & Medicine 2005;60(9):1987-2010. doi: <http://dx.doi.org/10.1016/j.socscimed.2004.08.056>.

9. Mackenbach JP. The persistence of health inequalities in modern welfare states: the explanation of a paradox. Social science & medicine 2012;75(4):761-9.

10. Backholer K, Sarink D, Beauchamp A, Keating C, Loh V, Ball K, Martin J, Peeters A. The impact of a tax on sugar-sweetened beverages according to socio-economic position: a systematic review of the evidence. Public health nutrition 2016;19(17):3070-84.

11. Escaron AL, Meinen AM, Nitzke SA, Martinez-Donate AP. Supermarket and Grocery Store–Based Interventions to Promote Healthful Food Choices and Eating Practices: A Systematic Review. Preventing Chronic Disease 2013;10:E50. doi: 10.5888/pcd10.120156.

12. Cameron AJ, Charlton E, Ngan WW, Sacks G. A systematic review of the effectiveness of supermarket-based interventions involving product, promotion, or place on the healthiness of consumer purchases. Current Nutrition Reports 2016;5(3):129-38.

13. Adam A, Jensen JD. What is the effectiveness of obesity related interventions at retail grocery stores and supermarkets? —a systematic review. BMC Public Health 2016;16:1247. doi: 10.1186/s12889-016-3985-x.

14. Hartmann-Boyce J, Bianchi F, Payne Riches S, Nourse R, Piernas C, Frie K, Jebb SA. Grocery store interventions to change food purchasing behaviours: protocol for a systematic review of randomized controlled trials. PROSPERO 2017;CRD42017068809:Available from: <http://www.crd.york.ac.uk/PROSPERO/display_record.php?ID=CRD42017068809>.

15. Higgins JPT, Green S. Cochrane Handbook for Systematic Reviews of Interventions Version 5.1.0 [updated March 2011] The Cochrane Collaboration, 2011. Available from wwwcochrane-handbookorg.

16. Ma Y, He FJ, Li N, Hao J, Zhang J, Yan LL, Wu Y. Salt sales survey: a simplified, cost-effective method to evaluate population salt reduction programs-a cluster-randomized trial. Hypertens Res 2015. doi: 10.1038/hr.2015.139.

17. Crockett RA, Hollands GJ, Jebb SA, Marteau TM. Nutritional labelling for promoting healthier food purchasing and consumption. Cochrane Database of Systematic Reviews 2011(9). doi: 10.1002/14651858.CD009315.

18. Thomas J, O'Mara-Eves A, Brunton G. Using qualitative comparative analysis (QCA) in systematic reviews of complex interventions: a worked example. Systematic reviews 2014;3:67. doi: 10.1186/2046-4053-3-67.

19. Brunton G, O'Mara‐Eves A, Thomas J. The ‘active ingredients’ for successful community engagement with disadvantaged expectant and new mothers: a qualitative comparative analysis. Journal of advanced nursing 2014;70(12):2847-60.

20. Ragin CC, Davey S. Fuzzy-Set/Qualitative Comparative Analysis 3.0. Irvine, California: Department of Sociology, University of California, 2016.

21. Lent MR, Vander Veur SS, McCoy TA, Wojtanowski AC, Sandoval B, Sherman S, Komaroff E, Foster GD. A randomized controlled study of a healthy corner store initiative on the purchases of urban, low-income youth. Obesity 2014;22(12):2494-500.

22. Phipps EJ, Braitman LE, Stites SD, Singletary SB, Wallace SL, Hunt L, Axelrod S, Glanz K, Uplinger N. Impact of a Rewards-Based Incentive Program on Promoting Fruit and Vegetable Purchases. American Journal of Public Health 2015;105(1):166-72.

23. Forwood SE, Ahern AL, Marteau TM, Jebb SA. Offering within-category food swaps to reduce energy density of food purchases: a study using an experimental online supermarket. International Journal of Behavioral Nutrition & Physical Activity 2015;12:85.

24. Huang A, Barzi F, Huxley R, Denyer G, Rohrlach B, Jayne K, Neal B. The effects on saturated fat purchases of providing internet shoppers with purchase- specific dietary advice: a randomised trial. PLoS Clinical Trials 2006;1(5):e22.

25. Winett RA, Moore JF, Wagner JL, Hite LA, Leahy M, Neubauer TE, Walberg JL, Walker WB, Lombard D, Geller ES. Altering shoppers' supermarket purchases to fit nutritional guidelines: an interactive information system. Journal of Applied Behavior Analysis 1991;24(1):95-105.

26. Anderson ES, Winett RA, Bickley PG, Walberg-Rankin J, Moore JF, Leahy M, Harris CE, Gerkin RE. The effects of a multimedia system in supermarkets to alter shoppers' food purchases: nutritional outcomes and caveats. Journal of Health Psychology 1997;2(2):209-23.

27. Ni Mhurchu C, Blakely T, Jiang Y, Eyles HC, Rodgers A. Effects of price discounts and tailored nutrition education on supermarket purchases: a randomized controlled trial. American Journal of Clinical Nutrition 2010;91(3):736-47.

28. Geliebter A, Ang IYH, Bernales-Korins M, Hernandez D, Ochner CN, Ungredda T, Miller R, Kolbe L. Supermarket discounts of low-energy density foods: effects on purchasing, food intake, and body weight. Obesity 2013;21(12):E542-8.

29. Ball K, McNaughton SA, Le HND, Gold L, Ni Mhurchu C, Abbott G, Pollard C, Crawford D. Influence of price discounts and skill-building strategies on purchase and consumption of healthy food and beverages: outcomes of the Supermarket Healthy Eating for Life randomized controlled trial. American Journal of Clinical Nutrition 2015;101(5):1055-64.

30. Budd N, Jeffries JK, Jones-Smith J, Kharmats A, McDermott AY, Gittelsohn J. Store-directed price promotions and communications strategies improve healthier food supply and demand: impact results from a randomized controlled, Baltimore City store-intervention trial. Public Health Nutrition 2017:1-11.

31. Kristal AR, Goldenhar L, Muldoon J, Morton RF. Evaluation of a supermarket intervention to increase consumption of fruits and vegetables. American Journal of Health Promotion 1997;11(6):422-5.

32. Waterlander WE, de Boer MR, Schuit AJ, Seidell JC, Steenhuis IHM. Price discounts significantly enhance fruit and vegetable purchases when combined with nutrition education: a randomized controlled supermarket trial. American Journal of Clinical Nutrition 2013;97(4):886-95.

33. Phipps EJ, Wallace SL, Stites SD, Uplinger N, Brook Singletary S, Hunt L, Axelrod S, Glanz K, Braitman LE. Using rewards-based incentives to increase purchase of fruit and vegetables in lower-income households: design and start-up of a randomized trial. Public Health Nutrition 2013;16(5):936-41.

34. Smith C, Parnell WR, Brown RC, Gray AR. Providing additional money to food-insecure households and its effect on food expenditure: a randomized controlled trial. Public Health Nutrition 2013;16(8):1507-15.

35. Brimblecombe J, Ferguson M, Chatfield MD, Liberato SC, Gunther A, Ball K, Moodie M, Miles E, Magnus A, Mhurchu CN, et al. Effect of a price discount and consumer education strategy on food and beverage purchases in remote Indigenous Australia: a stepped-wedge randomised controlled trial. The Lancet Public Health 2017;2(2):e82-e95.

36. Dhar SK, Hoch SJ. Price discrimination using in-store merchandising. Journal of Marketing 1996;60(1):17-30.

37. Epstein LH, Finkelstein E, Raynor H, Nederkoorn C, Fletcher KD, Jankowiak N, Paluch RA. Experimental analysis of the effect of taxes and subsides on calories purchased in an on-line supermarket. Appetite 2015;95:245-51.

38. Nederkoorn C, Havermans RC, Giesen JCAH, Jansen A. High tax on high energy dense foods and its effects on the purchase of calories in a supermarket. An experiment. Appetite 2011;56(3):760-5. doi: <https://doi.org/10.1016/j.appet.2011.03.002>.

39. Waterlander WE, Steenhuis IHM, de Boer MR, Schuit AJ, Seidell JC. Introducing taxes, subsidies or both: the effects of various food pricing strategies in a web-based supermarket randomized trial. Preventive Medicine 2012;54(5):323-30.

40. Waterlander WE, Steenhuis IHM, de Boer MR, Schuit AJ, Seidell JC. Effects of different discount levels on healthy products coupled with a healthy choice label, special offer label or both: Results from a web-based supermarket experiment. International Journal of Behavioral Nutrition and Physical Activity 2013;10 (no pagination)(59).

41. Waterlander WE, Steenhuis IHM, de Boer MR, Schuit AJ, Seidell JC. The effects of a 25% discount on fruits and vegetables: results of a randomized trial in a three-dimensional web-based supermarket. International Journal of Behavioral Nutrition & Physical Activity 2012;9:11.

42. Waterlander WE, Ni Mhurchu C, Steenhuis IHM. Effects of a price increase on purchases of sugar sweetened beverages. Results from a randomized controlled trial. Appetite 2014;78:32-9.

43. Dreze X, Hoch SJ, Purk ME. Shelf management and space elasticity. Journal of retailing 1994;70(4):301-26.

44. Foster GD, Karpyn A, Wojtanowski AC, Davis E, Weiss S, Brensinger C, Tierney A, Guo W, Brown J, Spross C, et al. Placement and promotion strategies to increase sales of healthier products in supermarkets in low-income, ethnically diverse neighborhoods: a randomized controlled trial. American Journal of Clinical Nutrition 2014;99(6):1359-68.

45. Thorndike AN, Bright O-JM, Dimond MA, Fishman R, Levy DE. Choice architecture to promote fruit and vegetable purchases by families participating in the Special Supplemental Program for Women, Infants, and Children (WIC): randomized corner store pilot study. Public Health Nutrition 2017;20(7):1297-305.

46. Jeffery RW, Pirie PL, Rosenthal BS, Gerber WM, Murray DM. Nutrition education in supermarkets: an unsuccessful attempt to influence knowledge and product sales. Journal of Behavioral Medicine 1982;5(2):189-200.

47. Russo JE, Staelin R, Nolan CA, Russell GJ, Metcalf BL. Nutrition Information in the Supermarket. Journal of Consumer Research 1986;13(1):48-70.

48. Elofsson K, Bengtsson N, Matsdotter E, Arntyr J. The impact of climate information on milk demand: Evidence from a field experiment. Food Policy 2016;58:14-23. doi: 10.1016/j.foodpol.2015.11.002.

49. Milliron B-J, Woolf K, Appelhans BM. A point-of-purchase intervention featuring in-person supermarket education affects healthful food purchases. Journal of Nutrition Education & Behavior 2012;44(3):225-32.

50. Wansink B, Soman D, Herbst KC. Larger partitions lead to larger sales: Divided grocery carts alter purchase norms and increase sales. Journal of Business Research 2017;75:202-9. doi: 10.1016/j.jbusres.2016.06.023.

51. Ball K, McNaughton SA, Le HN, Abbott G, Stephens LD, Crawford DA. ShopSmart 4 Health: results of a randomized controlled trial of a behavioral intervention promoting fruit and vegetable consumption among socioeconomically disadvantaged women. American Journal of Clinical Nutrition 2016;104(2):436-45.

52. Achabal DD, McIntyre SH, Bell CH, Tucker N. The Effect of Nutrition P-O-P Signs on Consumer Attitudes and Behavior. Journal of Retailing 1987;63(1):9.

1. NR: not reported; NS: not significant (where p values or confidence intervals not presented in study report) [↑](#footnote-ref-2)
2. Note, data here is that extracted from published studies; no additional calculation was conducted. Readers are encouraged to look to the full study reports for more information on outcome data (e.g. by individual arm or at different follow-up points). [↑](#footnote-ref-3)
3. For readers unfamiliar with QCA, we recommend reading Thomas et al to guide interpretation. Briefly: In crisp-set QCA each intervention scores 1 or 0 on each intervention characteristic included in the analysis to describe whether the intervention did or did not have the characteristic of interest. In our analysis these characteristics of interest were whether or not the intervention involved (1) an economic component, (2) changes to the physical store environment, (3) swaps, (4) consumer education/information, and whether or not (5) the intervention was based in a real grocery store. The outcome of our analysis was whether or not the intervention was associated with statistically significant changes in purchasing in the desired direction for at least one of the foods targeted by the intervention. Together these scores form an intervention’s configuration, which is thea set of conditions associated (or not associated) with statistically significant changes in the desired direction for at least one of the foods targeted by the intervention ; consistency represents the proportion of intervention versus control comparisons in each configuration which detected a significant difference in favour of the intervention for our primary outcome; solution coverage indicates the proportion of comparisons where an effect was detected that has one of the four configurations presented below, with raw coverage calculating the proportion explained by each configuration and unique coverage the proportion explained solely by that configuration. [↑](#footnote-ref-4)
4. The solution coverage of 0.862 indicates the proportion of comparisons eligible for QCA with an intervention associated with statistically significant changes in the desired direction for at least one of the foods targeted by the intervention that have one of the five configurations [↑](#footnote-ref-5)
5. The solution consistency of 0.892 gives the proportion of comparisons with one of the five configurations that is associated with statistically significant changes in the desired direction for at least one of the foods targeted by the intervention [↑](#footnote-ref-6)
6. The solution coverage of 0.857 indicates the proportion of comparisons eligible for QCA with an intervention that has the configuration and is not associated with statistically significant changes in the desired direction for at least one of the foods targeted by the intervention [↑](#footnote-ref-7)
7. The solution consistency of 0.923 gives the proportion of comparisons with either configuration that is not associated with statistically significant changes in the desired direction for at least one of the foods targeted by the intervention [↑](#footnote-ref-8)
